# Arginine methylation of PPP1CA by CARM1 regulates glucose metabolism and affects osteogenic differentiation and osteoclastic differentiation

**DOI:** 10.1002/ctm2.1369

**Published:** 2023-08-30

**Authors:** Lu Zhang, Guangjun Jiao, Yunhao You, Xiang Li, Jincheng Liu, Zhenqian Sun, Qinghui Li, Zihan Dai, Jinlong Ma, Hongming Zhou, Gang Li, Chunyang Meng, Yunzhen Chen

**Affiliations:** ^1^ Department of Spine Surgery Qilu Hospital of Shandong University Jinan Shandong China; ^2^ Department of Microorthopaedics Affiliated Hospital of Shandong University of Traditional Chinese Medicine Jinan Shandong China; ^3^ Department of Spine Surgery Affiliated Hospital of Jining Medical University Jining Shandong China; ^4^ Department of Orthopaedics The First Clinical College of Shandong University Jinan Shandong China; ^5^ Department of Spine Surgery Shandong Provincial Hospital Affiliated to Shandong First Medical University Jinan Shandong China; ^6^ Department of Spine Surgery Linyi Central Hospital Linyi Shandong China

**Keywords:** CARM1, glucose metabolism, osteoclast differentiation, osteogenic differentiation, osteoporosis

## Abstract

**Background:**

The imbalance between osteoblasts and osteoclasts may lead to osteoporosis. Osteoblasts and osteoclasts have different energy requirements, with aerobic glycolysis being the prominent metabolic feature of osteoblasts, while osteoclast differentiation and fusion are driven by oxidative phosphorylation.

**Methods:**

By polymerase chain reaction as well as Western blotting, we assayed coactivator‐associated arginine methyltransferase 1 (CARM1) expression in bone tissue, the mouse precranial osteoblast cell line MC3T3‐E1 and the mouse monocyte macrophage leukaemia cell line RAW264.7, and expression of related genes during osteogenic differentiation and osteoclast differentiation. Using gene overexpression (lentivirus) and loss‐of‐function approach (CRISPR/Cas9‐mediated knockout) in vitro, we examined whether CARM1 regulates osteogenic differentiation and osteoblast differentiation by metabolic regulation. Transcriptomic assays and metabolomic assays were used to find the mechanism of action of CARM1. Furthermore, in vitro methylation assays were applied to clarify the arginine methylation site of PPP1CA by CARM1.

**Results:**

We discovered that CARM1 reprogrammed glucose metabolism in osteoblasts and osteoclasts from oxidative phosphorylation to aerobic glycolysis, thereby promoting osteogenic differentiation and inhibiting osteoclastic differentiation. In vivo experiments revealed that CARM1 significantly decreased bone loss in osteoporosis model mice. Mechanistically, CARM1 methylated R23 of PPP1CA, affected the dephosphorylation of AKT‐T450 and AMPK‐T172, and increased the activities of phosphofructokinase‐1 and pructose‐2,6‐biphosphatase3, causing an up‐regulation of glycolytic flux. At the same time, as a transcriptional coactivator, CARM1 regulated the expression of pyruvate dehydrogenase kinase 3, which resulted in the inhibition of pyruvate dehydrogenase activity and inhibition of the tricarboxylic acid cycle, leading to a subsequent decrease in the flux of oxidative phosphorylation.

**Conclusions:**

These findings reveal for the first time the mechanism by which CARM1 affects both osteogenesis and osteoclast differentiation through metabolic regulation, which may represent a new feasible treatment strategy for osteoporosis.

## Graphical abstract

- CARM1 methylated R23 of PPP1CA, affected the dephosphorylation of AKT‐T450 and AMPK‐T172
- CARM1 increased the activities of phosphofructokinase‐1 (PFK1) and pructose‐2,6‐biphosphatase3 (PFKFB3), causing an up‐regulation of glycolytic flux
- CARM1 regulates glucose metabolism
- CARM1 affects both osteogenesis and osteoclast differentiation.

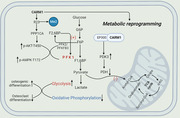

## 1 INTRODUCTION

The communication and coupling between osteoblasts and osteoclasts play a pivotal role in the process of bone remodelling. The imbalance of differentiation and functional coordination between the two types of cells is the basis of the pathogenesis of osteoporosis. Osteoblasts consume large amounts of adenosine triphosphate (ATP) during growth, differentiation and bone remodelling, and large energy requirements also occur during osteoclast differentiation from circulating monocyte precursors and fusion.^1^ The main source of energy for both types of cells is glucose. Glycolysis is a major metabolic pathway that meets ATP demand during osteoblast differentiation.^2^, ^3^ The differentiation and fusion of osteoclasts from circulating monocyte precursors is primarily driven by oxidative phosphorylation (OXPHOS).^4^, ^5^, ^6^ Proper energy metabolism processes are essential for the proper function of each cell in the bone remodelling unit.

Recently, incrementing attention has been given to the important functions and driving factors of metabolic reprogramming in osteoblast and osteoclast differentiation.^7^ When energy metabolism is impaired in bone cells, it can lead to disturbances in the balance of bone metabolism, causing the development of osteoporosis and other related diseases.^1^, ^8^ A number of signals or molecules capable of regulating cellular metabolism have been shown to regulate the function of osteoblasts and osteoclasts.^5^, ^9^ WNT‐LRP5 signalling facilitates bone formation by glucose metabolism reprogramming.^7^ Oestrogen reduces the number of osteoclasts by attenuating OXPHOS, thereby promoting mitochondrial apoptosis in early osteoclast progenitors.^10^ Thus, methods to regulate cellular energy metabolism may alter the progression of osteoporosis.^3^, ^11^, ^12^

Coactivator‐associated arginine methyltransferase 1 (CARM1) was ascertained at the time of its discovery as a coregulator of transcription.^13^, ^14^ Nevertheless, recent researches have disclosed novel roles for CARM1 in early development, autophagy and metabolism.^15^, ^16^, ^17^ As a type I protein arginine methyltransferase, CARM1 is capable of asymmetric dimethylation of protein, involving transcription factors, splicing factors and histones.^18^, ^19^, ^20^ Methylation of pyruvate kinase isoform M2 (PKM2) by CARM1 transforms the metabolism of breast cancer cells from OXPHOS to aerobic glycolysis.^21^ Nonetheless, whether CARM1 can regulate the energy metabolism of osteoblasts and osteoclasts is unclear.

Phosphofructokinase‐1 (PFK1), the rate‐limiting enzyme in the glycolysis process, catalyses the conversion of fructose 6‐phosphate (F‐6‐P) to fructose 1,6‐bisphosphate (F‐1,6‐BP).^22^ 6‐Phosphofructo‐2‐kinase (PFKFB3) catalyses the production of fructose‐2, 6‐diphosphate (F‐2, 6‐BP) from F6P, which is considered to be the strongest activator of PFK1.^23^ Pyruvate dehydrogenase kinase (PDK) is a family of four enzyme isoforms that phosphorylate and inhibit pyruvate dehydrogenase (PDH), a key enzyme in mitochondrial glucose oxidation,^24^, ^25^ PDK is one of the most important factors that can direct the carbon flux from OXPHOS to glycolysis.^26^, ^27^

Here, we identified CARM1 as a new factor that regulates osteogenesis and osteoclast differentiation through metabolic reprogramming. The results of animal experiments suggest that CARM1 can significantly reduce bone loss in osteoporosis model mice. Mechanistically, CARM1 methylates R23 of PPP1CA, which attenuates the dephosphorylation of AKT‐T450 and AMPK‐T172 and increases the activity of PFK1 and PFKFB3, causing an up‐regulation of glycolytic flux. At the same time, as a transcriptional coactivator, CARM1 improved the expression of PDK3, which resulted in the suppression of PDH activity and the tricarboxylic acid (TCA) cycle. Our data suggest that CARM1 coordinates osteogenic and osteoclastic differentiation by regulating metabolism, and CARM1 may be a new therapeutic target for osteoporosis in the future.

## 2 MATERIALS AND METHODS

### 2.1 Reagents and antibodies

The Seahorse XF Glycolytic Rate Assay Kit (103344‐100) and Seahorse XF Cellular Mitochondrial Stress Test Kit (103015‐100) were obtained from Agilent Technologies, and β‐glycerol disodium (G9422), ascorbic acid (A92902) and dexamethasone (D4902) were obtained from Sigma‒Aldrich. Recombinant mouse RANKL (CJ94) was obtained from NovoProtein Technology Co., Ltd. The PFK activity detection kit was obtained from Soleibao Technology Co., Ltd. DyLight 488 goat anti‐mouse IgG and DyLight 596 goat anti‐rabbit IgG were obtained from Abbkine Scientific. MK‐2206 dihydrochloride (GC16304) and SC79 (GC11645) were obtained from GLPBIO, and AICAR (Acadesine, S1802), Dorsomorphin (Compound C, S7840) and PFK15 (S7289) were obtained from Selleck. The Reactive Oxygen Species Assay Kit (S0033S), GSH and GSSG Assay Kit (S0053) and NADP+/NADPH Assay Kit (S0179) were obtained from Beyotime Biotechnology Ltd. CARM1 (NM_199141) Human Recombinant Protein and PPP1CA (NM_002708) Human Recombinant Protein were obtained from OriGene.

The information on the primary antibodies applied in this research are listed in Table S2.

### 2.2 Mice

C57BL/6 mice were bought from the Laboratory Animal Center of Shandong University (Jinan, China). The mice were placed in the Laboratory Animal Center of Shandong University Qilu Hospital and were fed in an air‐conditioned room at 23–25°C with a light–dark cycle time of 12 h, where they had ample access to water and food. All mice were divided into three groups randomly: the sham operation group, osteoporosis model group and osteoporosis model + *Carm1* lentivirus injection group (*n* = 6 for each group).

#### 2.2.1 Osteoporosis mouse model and lentivirus injection method

Eight‐week‐old female wild‐type C57BL/6 mice were anaesthetised with pentobarbital (50 mg/kg, intraperitoneal), and in the osteoporosis group, both ovaries were excised under sterile conditions. Mice in the sham group had part of the fat tissue around their ovaries removed.

Lentiviral intramedullary injection was administered 4 weeks after ovariectomy. As mentioned previously,^28^ intercondylar sulci of femur in mice were exposed under sterile conditions. Fifteen microlitres of CARM1 lentivirus (1 × 10^7^/mL) was injected into the bone marrow cavity using a Schickler's needle. Similarly, 15 μL (1 × 10^7^/mL) of NC lentivirus was injected into the femoral bone marrow cavity of mice in the osteoporosis group.

### 2.3 Bioluminescence imaging

After the mice were anaesthetised, d‐luciferin and sodium salt (MKBIO) (15 mg/mL) were injected through the tail vein at a dose of 10 μL/g. After 10 min, images were taken using a small animal live optical 3D imaging analysis system (PerkinElmer; IVIS Spectrum).

### 2.4 Bone dynamic analysis

Six weeks after lentivirus injection, mice were labelled with calcein (20 mg/kg; Sigma) through intraperitoneal injection at 10 and 3 days before being sacrificed. The non‐decalcified sections of femur were prepared, observed and photographed by fluorescence microscope (Olympus).

### 2.5 Micro‐CT

Mice were anaesthetised and then sacrificed. Femurs of mice were isolated and fixed in 4% paraformaldehyde. A microCT imaging system (PerkinElmer; Quantum GX2) was used to scan the femur tissue. The parameters were adjusted to a voltage of 90 kV, 88 μA, 14 min and a resolution of 36 μm pixel size. Images were reconstructed using Skyscan NRecon software, and sample parameters were analysed using CTVox software. When using CTVox software to analyse microCT data, we set the threshold value of bone trabeculae to >4500, and the area of interest was set in the epiphysis of the femoral shaft, and the line between the medial epicondyle and the lateral epicondyle of the femur extended to the proximal end for 200 μM. The 3D image of the bone trabeculae in the region of interest and the determination of each parameter were completed by CTVox software.

### 2.6 Bone histomorphometry and immunohistochemistry

All procedures were carried out as reported previously.^29^

### 2.7 Lentivirus‐mediated overexpression and knockout

The *Carm1*‐ and *Ppp1ca*‐knockout (KO) lentiviruses based on CRISPR/Cas9 technology were provided by Shanghai Genechem Co., Ltd. *Carm1*‐overexpressing lentivirus (luciferase vector) was bought from the GenePharma Corporation. According to the reviewer's opinion, our newly ordered lentivirus construction strategy is as follows: mRunx2‐promoter‐NM_021531‐3flag‐T2A‐firefly_Luciferase‐IRES‐Puromycin. For virus transfection, 80% confluent cells were incubated with lentivirus and polybrene (final concentration 8 μg/mL), and then the medium was changed to complete medium after 12 h. After 72 h, puromycin (Biosharp) was used for screening, and target gene expression was verified by Western blot.

### 2.8 SiRNA and plasmid transfection

SiRNA targeting *Pdk3* and scrambled siRNA (GenePharma) were diluted with DEPC water. For a single well of a six‐well plate, 100 pmol siRNA and 5 μL Lipofectamine 2000 (11668019; Thermo Fisher Scientific) were added to 250 μL Opti‐MEM I (31985070; Thermo Fisher Scientific) mixed thoroughly and placed at room temperature for 5 min. For transfection, the culture medium was replaced with 1.5 mL Opti‐MEM I, and the transfection mixture was added. The transfection complexes were removed after 4 h and replaced with fresh media. Analysis of knockdown efficiency and other experiments were conducted 48 h post‐transfection.

For plasmid transfection, the procedure was the same as siRNA transfection. The amount of plasmid and Lipofectamine 2000 was 4 μg and 10 μL, respectively. Plasmids overexpressing *Carm1*, *Ppp1ca*‐WT, *Ppp1ca*‐R23,142,317K, *Ppp1ca*‐R23K, *Ppp1ca*‐R142K, *Ppp1ca*‐R317K, *PPP1CA*‐WT, *PPP1CA*‐R23,142,317K, *PPP1CA*‐R23K, *PPP1CA*‐R142K and *PPP1CA*‐R317K were synthesised by GenePharma Corporation.

### 2.9 Quantitative real‐time polymerase chain reaction

Quantitative real‐time polymerase chain reaction (qRT‐PCR) was performed as previously described.^30^

### 2.10 Western blotting and co‐immunoprecipitation

Western blotting was performed based on previous studies.^31^ The obtained PVDF membrane was incubated with primary antibody overnight at 4°C. Next, PVDF membranes were incubated in peroxidase‐conjugated avidin goat anti‐rabbit IgG or goat anti‐mouse IgG (ZSGB‐BIO) (1:5000) for 1 h at room temperature. Membranes were then scanned, and protein levels were normalised to β‐actin (1:1000) as a control. A Tanon‐5200 chemiluminescence imaging system (Shanghai, China) and ImageJ software (NIH) were used to record and quantify signal intensities.

For co‐immunoprecipitation (Co‐IP), whole‐cell extracts were prepared with Pierce IP lysis buffer (87787; Thermo Scientific). Then, the extract was incubated with the corresponding antibody overnight at 4°C. Protein A&G beads (Bersinbio) were added and incubated at 4°C for 4 h. The coprecipitated proteins were washed with SDS loading buffer for 5 min at 95°C. Subsequent results were obtained by Western blotting as described above.

### 2.11 Immunofluorescence

All procedures were carried out as previously reported,^32^ and images were taken with a Dragonfly 200 high‐speed confocal platform (Andor).

### 2.12 Measurement of extracellular acidification rate

The extracellular acidification rate (ECAR) was measured in an XF96 extracellular analyser (Seahorse; Agilent). MC3T3‐E1 (1 × 10^4^ cells per well) and RAW264.7 (2 × 10^4^ cells per well) cells were seeded into 96‐well plates. On the next day, the media was changed to analysis media containing 10 mM glucose, 1 mM pyruvate and 2 mM glutamine. The cells were incubated in a CO_2_‐free incubator at 37°C for 1 h. Cells were sequentially exposed to Rot/AA (0.5 μM) and 2‐DG (50 mM). Data were processed using Wave software (Agilent).

### 2.13 Measurement of oxygen consumption rate

The oxygen consumption rate (OCR) was measured in an XF96 extracellular analyser (Seahorse; Agilent). Cells were treated as described above. Cells were sequentially exposed to oligomycin (1 μM), FCCP (1 μM) and rotenone (0.5 μM).

### 2.14 In vitro methylation assays

An in vitro methylation assay was performed as previously described.^32^ The in vitro methylation reaction was performed in 30 μL methylation buffer (50 mM Tris pH 8.5, 20 mM KCl, 10 mM MgCl_2_, 1 mM β‐mercaptoethanol, and 100 mM sucrose) containing 3 μg His‐PPP1CA, 3 μg CARM1 and 3 μM S‐adenosyl methionine (SAM) (Sigma; A4377). Methylation reactions were incubated at 30°C for 1.5 h. The reaction system was stopped by adding 5× SDS loading buffer and was resolved by SDS‐PAGE. Mass spectrometry analysis of PPP1CA arginine methylation was performed by AIMSMASS Co., Ltd.

### 2.15 In vitro protein–protein interaction assay

Wild‐type and mutant PPP1CA were translated by the T7 Quick Coupled Translation/Transcription system (Promega). Interaction with Myc‐CARM1 fusion protein was conducted as described above. The methyl group is provided by adenosyl‐l‐methionine, S‐[methyl‐^3^H] (SAM[^3^H]) (1 mCi/mL stock solution; PerkinElmer). The separated samples were exposed to X‐ray film.

### 2.16 Liquid scintillation counting

The PPP1CA (WT and mutant)‐CARM1‐SAM[3H] reaction system was stopped by adding 5× SDS loading buffer and resolved by SDS‐PAGE. After electrophoresis, PPP1CA (wild‐type and mutant) strips were removed, and proteins in the strips were extracted with a Micro Protein Page Recovery Kit (Sangon Biotech). Then, scintillation solution was added to the protein, and the proteins were counted using a 1450 LSC & Luminescence Counter (PerkinElmer).

### 2.17 Statistics and reproducibility

Statistical testing was performed using the unpaired two‐tailed Student's *t*‐test and two‐way ANOVA. All data are shown as the mean ± standard deviation (SD). All experiments were repeated at least three times unless otherwise indicated. A *p* value < .05 was considered statistically significant.

### 2.18 Ethics approval

This experimental plan was approved by the Medical Ethics Committee of Shandong University Qilu Hospital (Approval No.: 2021156). All human bone samples used in the experiment came from surgically removed bone tissue, and the collection of bone specimens did not have any impact on the treatment. The animal experiments were approved by the Animal Ethics Committee of Qilu Hospital of Shandong University (Approval number: Dull‐2021‐054), and all animal experiments were conducted to minimize animal suffering.

## 3 RESULTS

### 3.1 CARM1 promotes osteoblast differentiation of primary mouse osteoblasts and impairs osteoclast differentiation of mouse bone marrow‐derived macrophage

**FIGURE 1:**
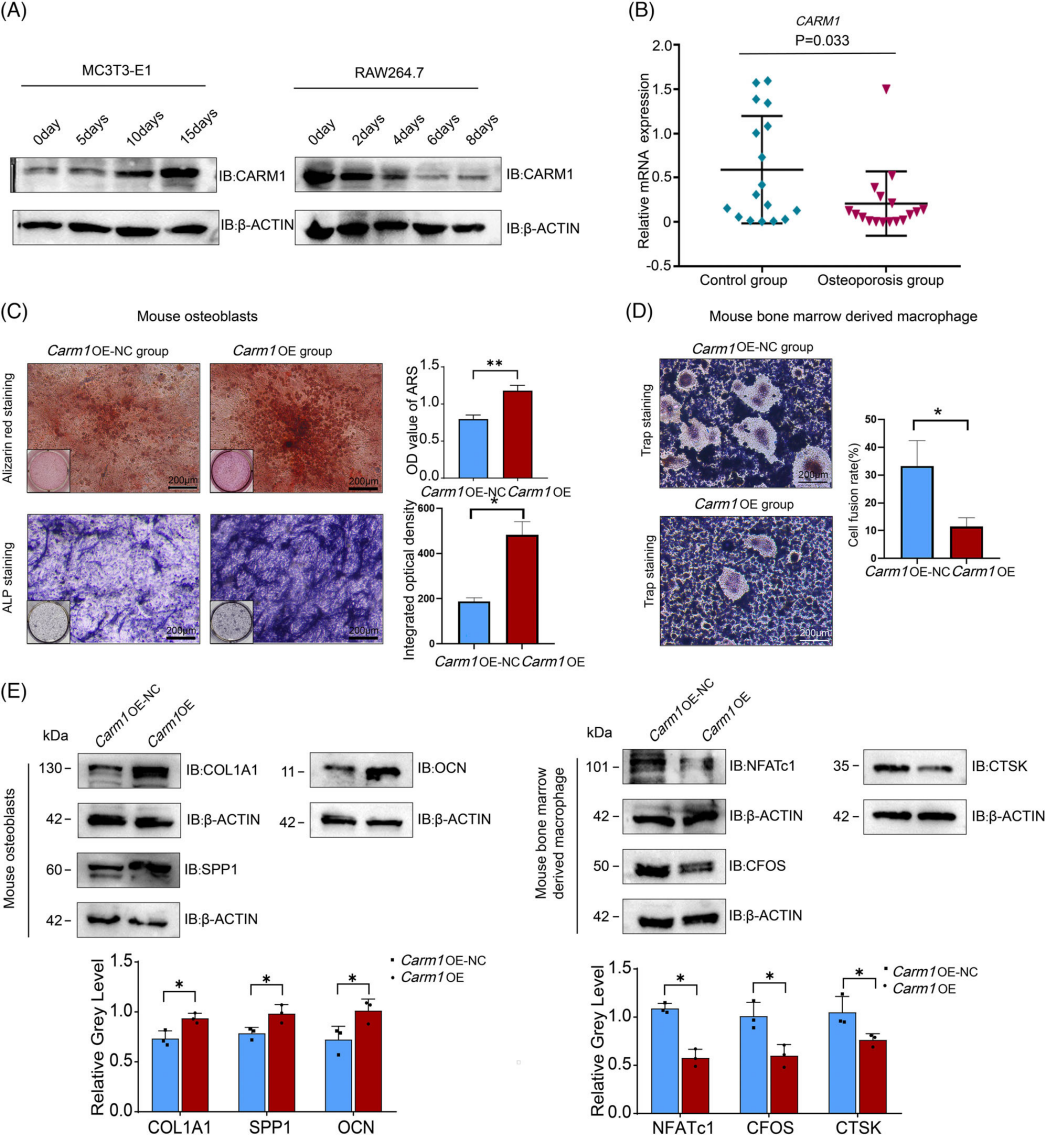
CARM1 promotes osteoblast differentiation of primary mouse osteoblasts and impairs osteoclast differentiation of mouse bone marrow‐derived macrophage (A) Western blot analysis of the expression of CARM1 during osteogenic differentiation and osteoclastic differentiation. (B) Quantification analysis of CARM1 expression in bone samples from patients with osteoporosis. (C) Representative images of ARS and ALP staining in mouse osteoblasts Carm1‐OE and NC cells. (D) Representative images of TRAP staining in BMDM Carm1‐OE and NC cells after osteoclast induction. (E) Western blot analysis of osteogenesis‐related and osteoclast‐related genes expression, and quantitative data analysis of Western blot results. ** represents *p* < .01 vs. other groups, * represents *p* < .05 vs. other groups.

To clarify the coordinated character of CARM1 in bone resorption and bone formation, we first searched and analysed the data related to osteoporosis and osteogenic and osteoclastic differentiation in the GEO database. The GSE156508 data^33^ showed that *CARM1* expression was down‐regulated in primary osteoblasts from osteoporotic fracture patients (Figure S1a). Another dataset, GSE176265,^34^ suggested that *Carm1* was down‐regulated during osteoclast differentiation (Figure S1b). Western blotting showed that the expression of CARM1 increased during osteogenic differentiation and decreased during osteoclastic differentiation (Figure 1a). We used RT‐PCR to detect *CARM1* expression in bone samples from patients with osteoporosis, and *CARM1* expression was down‐regulated in the osteoporosis group compared with the control group (Figure 1b). To clarify whether CARM1 regulates osteogenesis and osteoblastic differentiation, overexpression (OE) of Carm1 was achieved by transfection of the plasmid in mouse osteoblasts and mouse bone marrow‐derived macrophage (BMDM); meanwhile, we performed a CRISPR‒Cas9‐based screen to KO *Carm1* in the mouse precranial osteoblast cell line MC3T3‐E1 and the mouse monocyte macrophage leukaemia cell line RAW264.7 and stably overexpressed *Carm1* by transfection with lentivirus (Figures S2a and b). Next, we explored the role of CARM1 in osteogenic differentiation of MC3TE‐E1 cells. The cells in the *Carm1* OE group and negative control (NC) group were grown in osteogenic medium for 0, 5, 10 and 15 days. qRT‐PCR showed that osteogenic induction in MC3TE‐E1 cells up‐regulated osteogenic‐related genes, including osteocalcin (*Ocn*), bone bridging protein (*Spp1*) and α1‐1 collagen (*Col1a1*), in a time‐dependent manner. (Figure S1c). In addition, results of ALP staining and Alizarin Red S (ARS) staining showed that ALP activity and extracellular matrix mineralisation were also significantly elevated in *Carm1*‐overexpressing mouse osteoblasts (Figure 1c). The Western blot results showed that *Carm1* OE improved osteogenesis‐related gene expression (Figure 1e). The same results were confirmed in MC3T3‐E1 cells (Figures S1e, f, h and S2c)

Next, we explored the function of CARM1 in osteoclast differentiation. *Carm1*‐OE and NC BMDM and RAW264.7 cells were cultured in osteoclastic medium. The RT‐PCR results showed that with increasing induction time, osteoclastic genes, including cathepsin k (*Ctsk*), Fos proto‐oncogene, AP‐1 transcription factor subunit (*C‐fos*) and nuclear factor of activated T‐cell cytoplasmic 1 (*Nfatc1*), were significantly down‐regulated in the *Carm1*‐OE group (Figure S1d). TRAP staining demonstrated the suppression of *Carm1*‐OE on osteoclast differentiation (Figure 1d). The same results were observed in Western blot experiments (Figure 1e). In addition, we once again verified the function of CARMI in RAW264.7 cells (Figures S1j, h, i, j and S2d, e). Collectively, these results demonstrated that CARM1 promoted osteogenic differentiation and impaired osteoclastic differentiation.

### 3.2 CARM1 supplementation reduces bone loss and enhances osteogenesis in osteoporosis model mice

**FIGURE 2:**
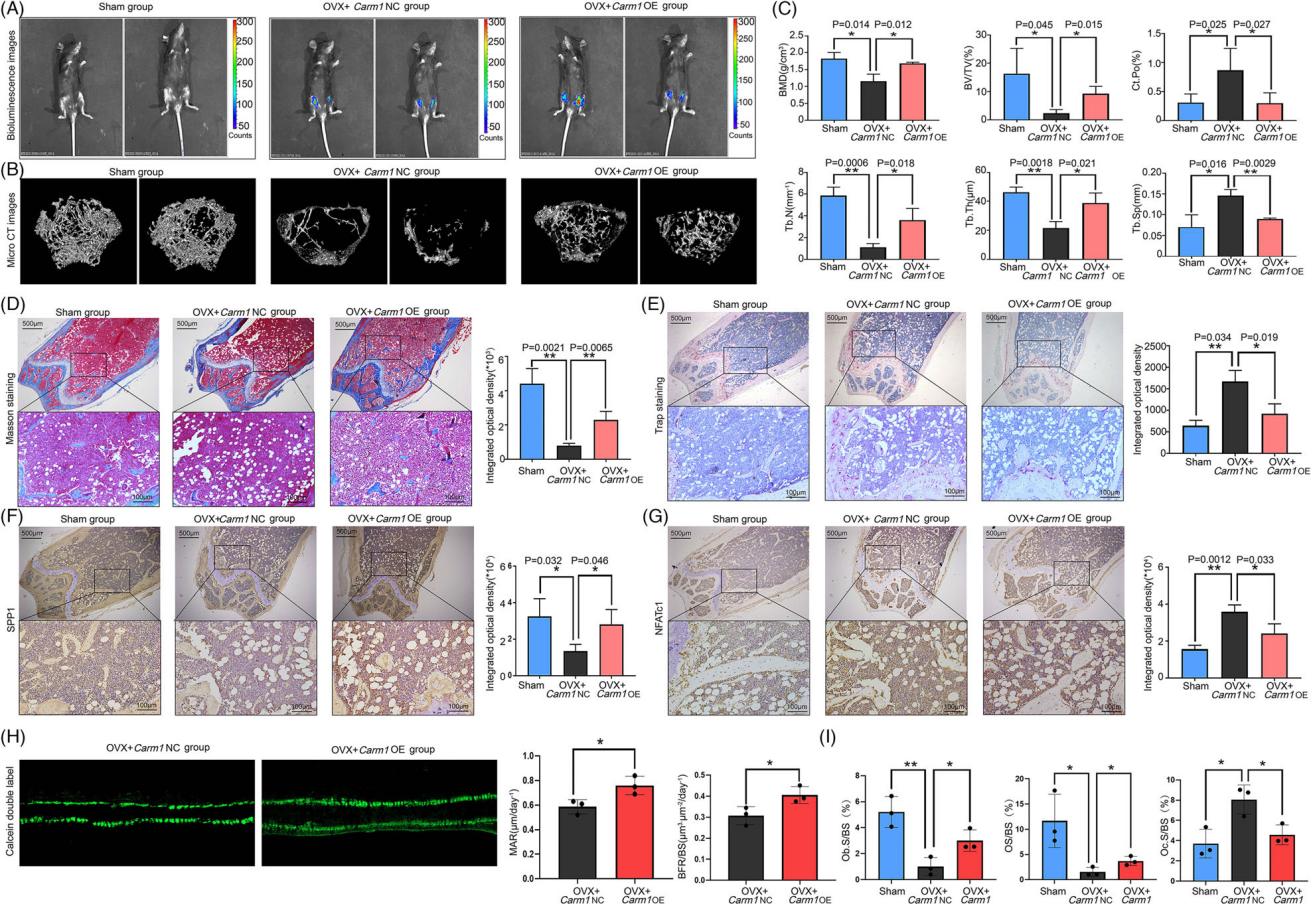
Carm1 supplementation reduces bone loss and enhances osteogenesis in ovariectomised mice.(A) Bioluminescence images of mice after the intramedullary injection of lentivirus. (B) Representative micro‐CT images of trabecular bone from the femoral metaphysis of mice from the sham‐operated group, NC group and Carm1‐OE group. (C) Trabecular bone mineral density (BMD, g/cm3), cancellous bone volume (BV/TV, %), trabecular thickness (Tb.Th), total cross‐sectional cortical bone area (B.Ar), trabecular number (Tb.N), trabecular separation (Tb.Sp) and d cortical porosity (Ct. Po) were determined by micro‐CT analysis. (D) Representative image of Masson's trichrome staining of mouse femur sections. (E) TRAP staining of distal metaphysis and diaphyseal cortical bone of the femurs. (F) Immunohistochemical staining of osteogenesis‐related genes in mouse femur sections. (G) Immunohistochemical staining of osteoclast‐related genes in mouse femur sections. (H) Calcein double label staining image and quantitative analysis of MAR and BFR in NC group and Carm1‐OE group mice. (I) Quantitative analysis results of multinucleated osteoclast number/bone surface (N. Oc/BS), osteoid surface (OS/BS ES) and osteoblast surface/millimetre of bone perimeter (Ob. S/BS). * represents *p* < .05 vs. other groups, ** represents *p* < .01 vs. other groups.

To assess the potential function of CARM1 in bone loss in oophorectomy mice, 10‐week‐old osteoporosis model mice were adopted intramedullary injection^28^, ^35^ of *Carm1*‐overexpressing or control lentivirus (pLV‐Luci vector) starting 1 day after oophorectomy. The bioluminescence results showed that the NC group and the *Carm1*‐OE group were locally infected with lentivirus in the femurs of mice after intramedullary injection (Figure 2a). RT‐qPCR results also confirmed the OE of *Carm1* in femoral tissues (Figure S3d). Microquantitative computed tomography (micro‐CT) analysis showed that *Carm1*‐OE could reduce ovariectomy‐induced bone loss (Figure 2b). Further analysis of trabecular bone in distal femoral metaphysis by micro‐CT showed that the bone mineral density (BMD), cancellous bone volume/tissue volume (BV/TV), trabecular thickness (Tb.Th) and trabecular number (Tb.N) of *Carm1*‐OE mice were higher than those of the NC group; in contrast, trabecular separation (Tb.Sp) and cortical porosity (Ct. Po) decreased (Figure 2c). Haematoxylin–eosin staining showed that there was significantly less trabecular bone in the NC group than in the sham‐operated group and *Carm1*‐OE group (Figure S2b). We repeated the above experiments with *Carm1‐*OE lentivirus with Runx2 promoter, confirming CARM1's role in promoting bone formation in vivo (Figure S3a, b, and c).

Bone formation and resorption levels were also assessed in ovariectomised mice of each group. Masson staining was used to assess bone collagen levels, it was found that the *Carm1*‐OE group had more bone collagen in the femurs (Figure 2d). However, compared with the sham‐operated group and *Carm1*‐OE group, TRAP staining showed that there were more osteoclasts in the cortex and trabecular bone of the NC group (Figure 2e). Immunohistochemical staining results of femoral sections showed that compared with the NC group, OCN and SPP1 were up‐regulated in the *Carm1*‐OE group (Figures 2f and S2f), while the osteoclast genes, CTSK, RANKL and NFATc1 were significantly down‐regulated (Figures 2g and S2g). The results of calcein double labelling experiment confirmed that CARM1 promoted bone formation in osteoporosis model mice in vivo, and the mineral apposition rate (MAR) and bone formation rate (BFR) of mouse bone samples in *Carm1*‐OE group were higher than those in control group. Quantitative analysis results of multinucleated osteoclast number/bone surface (N. Oc/BS), osteoid surface (OS/BS ES) and osteoblast surface/millimetre of bone perimeter (Ob. S/BS) showed that osteoclast was inhibited and osteogenesis was relatively active in *Carm1*‐OE group. In brief, the results indicated that CARM1 supplementation can reduce bone loss and stimulate bone formation to a certain extent in osteoporosis model mice.

### 3.3 CARM1 mediates metabolic reprogramming in osteoblasts and osteoclasts and regulates glycolytic flux by increasing PFK1 and PFKFB3 activity

**FIGURE 3:**
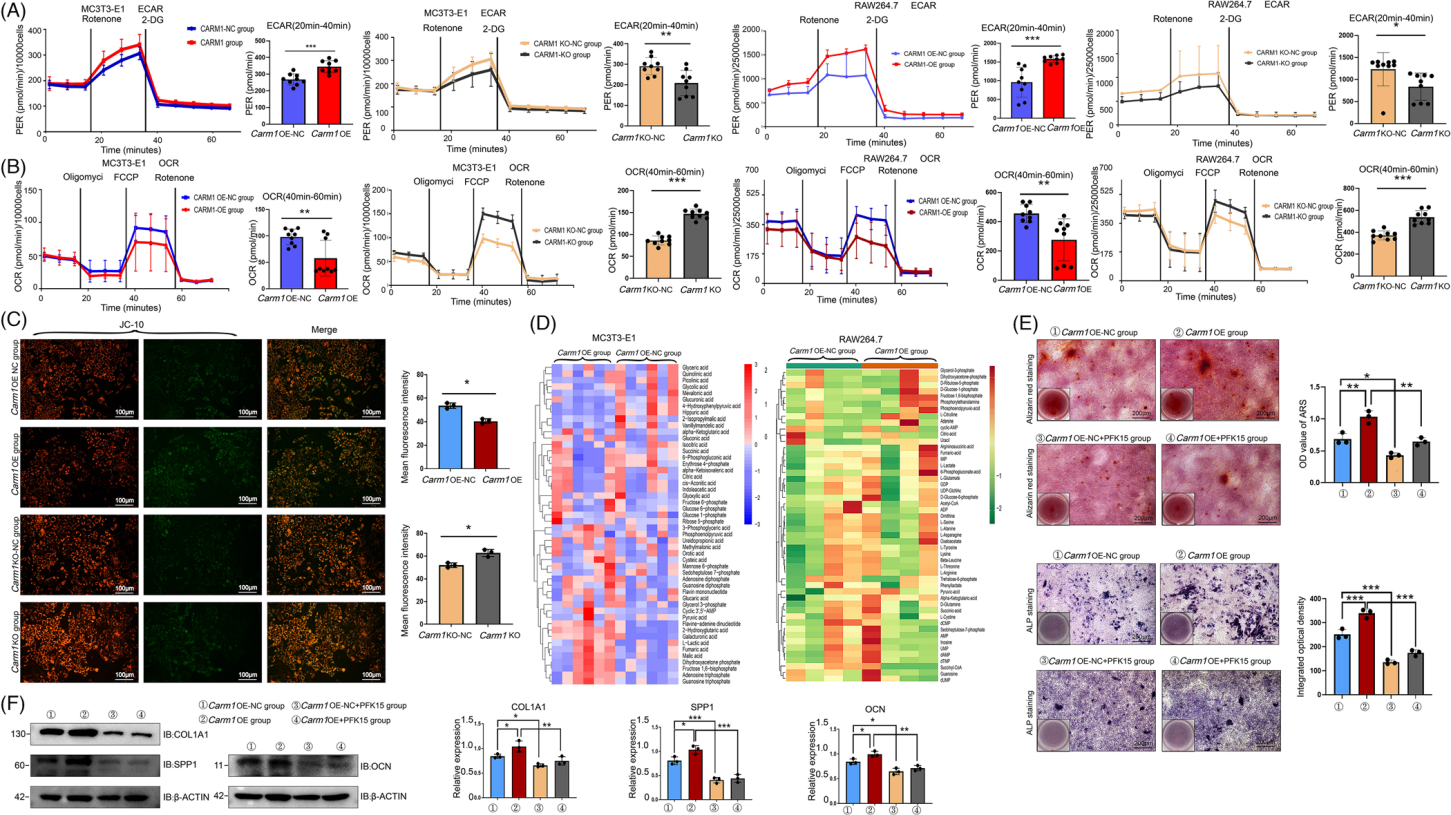
Carm1 mediates metabolic reprogramming in osteoblasts and osteoclasts and regulates glycolytic flux by increasing PFK1 and PFKFB3 activity. (A) The ECAR curves in Carm1‐OE/KO and NC cells treated with rotenone/antimycin A and 2‐DG (*n* = 4 independent experiments). (B) The OCR curves in Carm1‐OE/KO and NC cells treated with oligomycin, FCCP and rotenone/antimycin A (*n* = 4 independent experiments). (C) Representative images of JC‐10 staining of Carm1‐OE/KO and NC cells. (D) Heatmap of metabolomic assays for the Carm1‐OE/KO and NC groups of MC3T3‐E1 and RAW264.7 cells. (E) Representative images of ARS and ALP staining in MC3T3‐E1 cells after treatment with PFK15. (F) Western blot analysis of the expression of osteogenesis related genes in MC3T3‐E1 cells after treatment with PFK15.

We next investigated whether CARM1 regulates metabolic fluxes. Previous researches have indicated that CARM1 functions as a monitor of cellular glucose metabolism and is capable of reprogramming OXPHOS to aerobic glycolysis.^21^, ^36^ In MC3T3‐E1 and RAW264.7 cells, *Carm1* OE significantly elevated the ECAR (Figures 3a and S4a). Moreover, *Carm1* KO significantly increased the cell OCR and reduced lactate (Figures 3b and S4b). The mitochondrial membrane potential (△*Ψ*_m_) measurements also suggested that △*Ψ*_m_ in the *Carm1*‐KO group was higher than that in the NC group (Figures 3c and S5c).

To clarify the specific mechanism of CARM1 regulation of glucose metabolism, metabolomics analysis was performed on MC3T3‐E1 and RAW264.7 cells transfected with *Carm1*‐OE and NC lentivirus. Metabolomic analysis revealed that fructose 1,6‐diphosphate was the only metabolite up‐regulated in both cell lines in the *Carm1*‐OE group compared with the NC group (Figures 3d and S5a, b). These results suggested that the activity of PFK1 was up‐regulated by CARM1 in MC3T3‐E1 and RAW264.7 cells. In the PFK1 activity assay, we confirmed this conclusion, and PFK1 activity in the CARM1‐OE group was significantly higher than that in the control group (Figure S5b). PFKFB3 catalyses the synthesis of F‐2,6‐BP and promotes glycolytic flux with its high kinase activity. PFK15 is a highly effective and selective inhibitor of PFKFB3.^37^ The effect of CARM1 in promoting osteogenic differentiation could be reversed by PFK15 (Figures 3e and S4c). Results of Western blot indicated that the expression changes in genes related to osteogenesis induced by CARM1 were restored after PFK15 treatment (Figures 3f and S4d).

### 3.4 Activation of PFK1 and PFKFB3 is due to phosphorylation of AKT and AMPK

**FIGURE 4:**
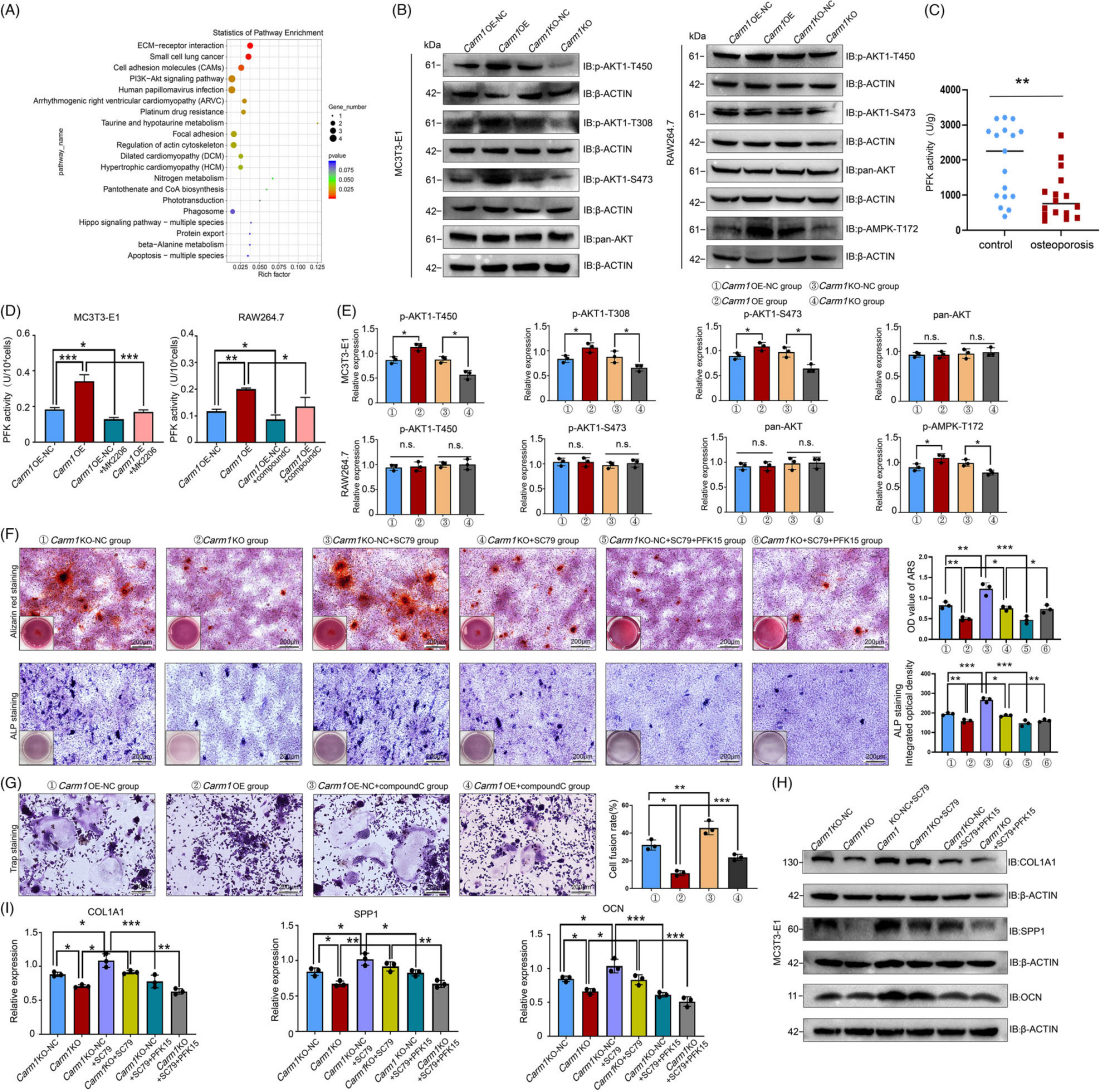
Activation of PFK1 and PFKFB3 is due to phosphorylation of AKT and AMPK. (A) KEGG enrichment analysis of the signalling pathways correlated with the differentially expressed mRNAs between MC3T3‐E1 Carm1‐OE and NC cells. (B) Western blot analysis of AKT and phosphorylated AKT expression in MC3T3‐E1 Carm1‐OE/KO and NC cells, Western blot analysis of expression of AKT/AMPK and phosphorylated AKT/AMPK in RAW264.7 Carm1‐OE /KO and NC cells. (C) Results of PFK activity in bone samples of osteoporosis group and control group. (D) Analysis of PFK activity data after MK2206 and Compound C application in MC3T3‐E1 and RAW264.7 cells. (E) Quantitative data analysis of Western blot results. (F) Representative images of ARS and ALP staining in MC3T3‐E1 cells after treatment with SC79 and PFK15. (G) Representative images of TRAP staining in RAW264.7 cells after treatment with compound C. (H) Western blot analysis of the expression of osteogenesis‐related genes in MC3T3‐E1 cells after treatment with SC79 and PFK15. (I) Quantitative data analysis of Western blot results. * represents *p* < .05 vs. other groups, ** represents *p* < .01 vs. other groups.

Studies by Liu et al.^21^ and Wang et al.^38^ revealed proteins that can interact directly with CARM1 and proteins with arginine methylation sites, but PFK1 and PFKFB3 were not among these proteins. AKT and AMPK are able to activate PFK1 and PFKFB3 when they exert kinase activity,^22^, ^39^, ^40^ and both kinases can be regulated by CARM1.^41^, ^42^ To decipher the molecular mechanisms by which CARM1 regulates cellular metabolism, transcriptomic assays were performed on *Carm1*‐OE MC3T3E1 cells (Figure S4a), and the results showed that the osteogenesis‐related genes *Spp1* and *Ocn* and several members of the *Col1* family had up‐regulated expression in the *Carm1*‐OE group. Subsequent Kyoto Encyclopedia of Genes and Genomes (KEGG) analysis showed that the PI3K–AKT signalling pathway was one of the main enriched pathways with differentially expressed genes (Figure 4a). Western blot results demonstrated that *Carm1* OE up‐regulated AKT phosphorylation, including Thr450, Thr308 and Ser473, in MC3T3‐E1 and mouse osteoblast cells (Figures 4b, d and S7a). However, in RAW264.7 cells, which do not show such regulation as described above, the kinase with altered phosphorylation levels is AMPK‐Thr172 (Figure 4b and d). Interestingly, changes in the phosphorylation levels of PFK1 and PFKFB3 were observed in both cell lines (Figure S6b and c).

Next, we tested whether the regulation of PFK enzyme activity and osteogenic and osteoclastic differentiation by CARM1 could be reversed by inhibitors or activators of AKT and AMPK. The PFK activity test showed that PFK activity decreased in bone samples from patients with osteoporosis (Figure 4c), and in mc3t1‐e1 and RAW264.7 cells, the regulation of PFK activity by CARM1 could be reversed by MK2206 (an AKT inhibitor) and Compound C (an AMPK inhibitor), respectively (Figure 4d). ALP staining and ARS staining results showed that MK2206 prevented CARM1 from promoting osteoblastic differentiation (Figure S6e). The inhibition of osteogenic differentiation caused by *Carm1* KO was rescued by SC79 (an AKT activator), and PFK15 reversed this modulation again (Figure 4f). The inhibition of osteoclastic differentiation by *Carm1* OE was rescued by Compound C during osteoclast induction (Figure 4g). The expression of osteoblast‐related genes, subject to regulation by CARM1, was also affected by AKT inhibitors and activators (Figures 4h, i, S6e, f and S7c).

### 3.5 CARM1 methylates PPP1CA at R23/142/317

**FIGURE 5:**
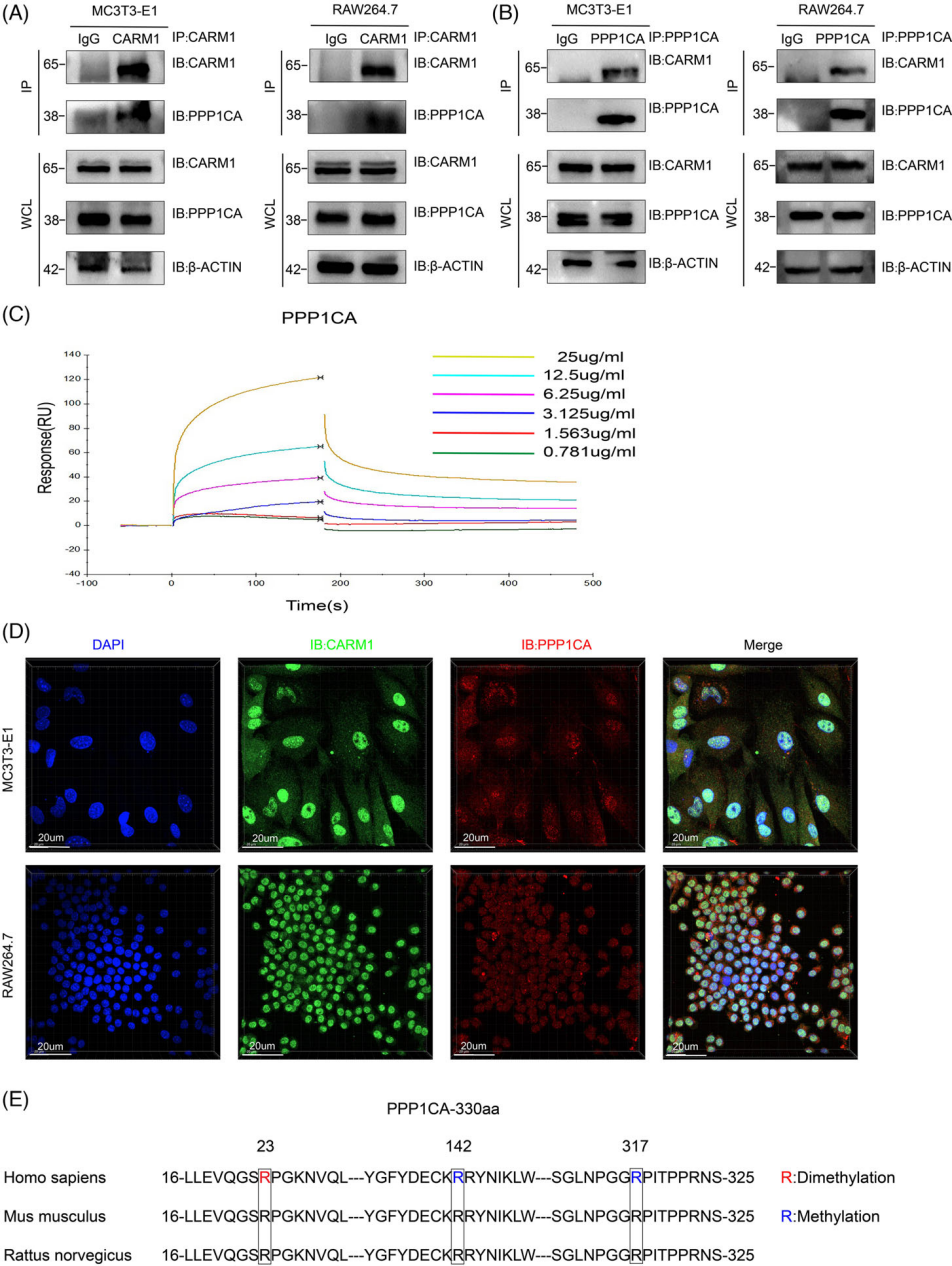
CARM1 methylates PPP1CA at R23/142/317 (A) Co‐immunoprecipitation of CARM1 with endogenous PPP1CA in MC3T3‐E1 and RAW264.7 cells. (B) Co‐immunoprecipitation of PPP1CA with endogenous CARM1 in MC3T3‐E1 and RAW264.7 cells. (C) The results of the Biacore assay for the binding strength of PPP1CA to CARM1. (D) Representative images of immunofluorescence staining of PPP1CA and CARM1 in MC3T3‐E1 and RAW264.7 cells. (E) Sequence of the evolutionarily conserved residues R23, R142, and R317 in PPP1CA. R (red): dimethylation site detected by in vitro methylation assay; R (blue): single methylation sites detected by in vitro methylation assay.

Dephosphorylation of AKT‐Thr308 depends on the regulation of protein phosphatase 2A (PP2A).^43^ Nevertheless, the phosphorylation of AKT‐Thr450 is regulated by PP‐1alpha^44^ (PPP1CA), a functional subunit of protein phosphatase 1 (PP1). Simultaneously, AMPK‐Thr172 is also dephosphorylated by PP1. PPP1CA is presumed to be an intermediate between AKT and AMPK regulated by CARM1. In the study by Liu et al., the results of GST pulldown showed that CARM1 was able to bind PPP1CA (Table S1).^21^ Next, the direct binding of CARM1 and PPP1CA was verified. Co‐IP of CARM1 in MC3T3‐E1 and RAW264.7 cells indicated that CARM1 interacted with PPP1CA (Figure 5a). Co‐IP of PPP1CA led to the same conclusion (Figure 5b). The binding relationship between the two proteins was again verified in the HEK‐293T cell line (Figure 5c). Myc‐CARM1 was coupled to a CM5 chip, and His‐PPP1CA protein was used as the mobile phase for the Biacore assay, which showed that the binding strength increased with increasing protein concentration (Figure 5d).

Then, cellular immunofluorescence staining was performed to clarify the intracellular distribution of CARM1 and PPP1CA. Confocal microscopy images showed colocalisation of CARM1 and PPP1CA in the cytoplasm and nucleus (Figure 5e). PPP1CA is highly conserved during evolution, and the amino acid sequence of PPP1CA is consistent among different species (Figure 5f). In vitro methylation assays were used to clarify the arginine methylation site of PPP1CA. In the presence of purified CARM1 and S‐adenosylmethionine, three arginine residues (R23, R142 and R317) were identified using liquid chromatography with tandem mass spectrometry (Figures S8a, b, c, and d).

### 3.6 PPP1CA R23 methylation inhibits the dephosphorylation of AKT‐Thr450 and AMPK‐Thr172

**FIGURE 6:**
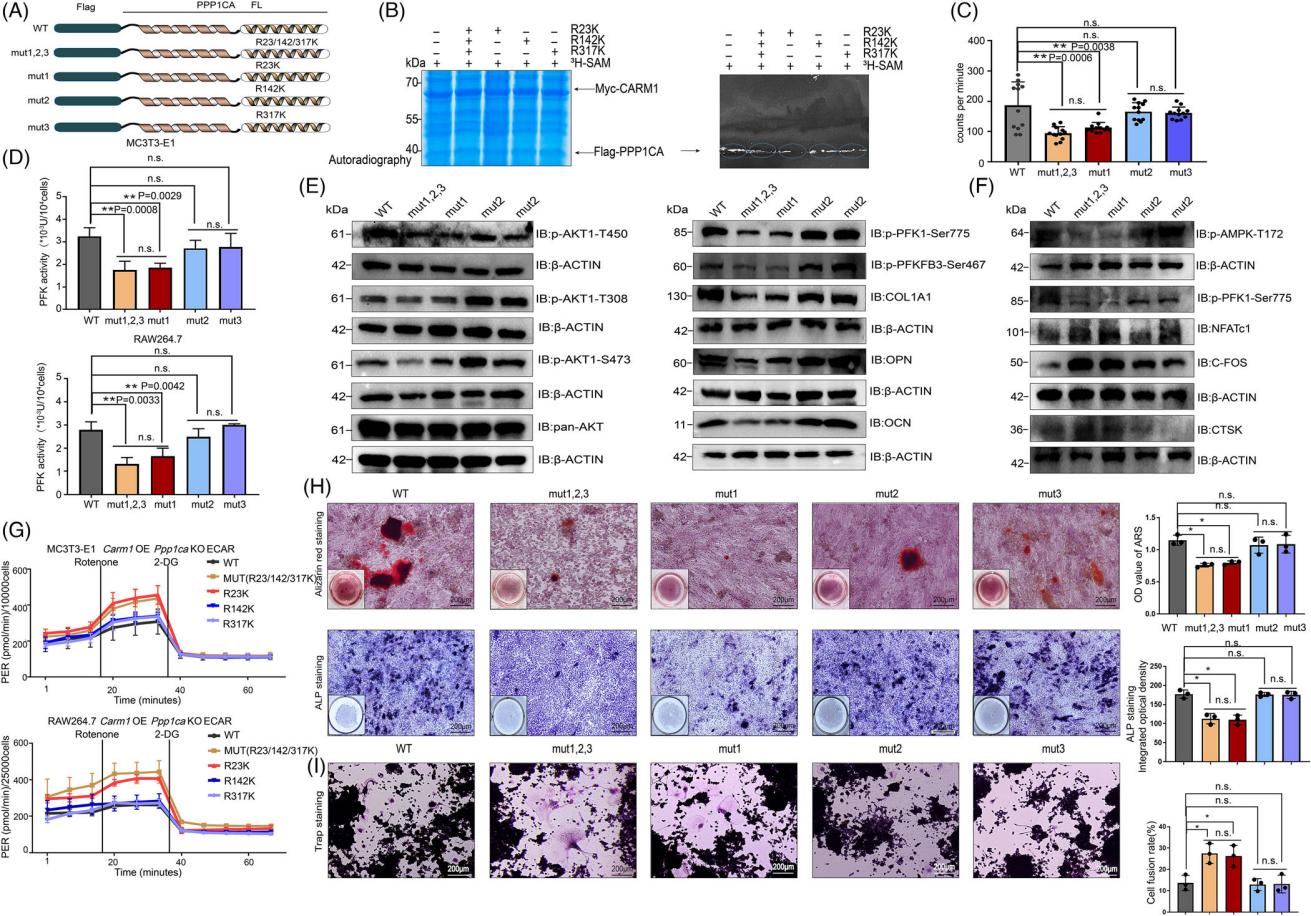
R23 methylation inhibits the dephosphorylation of AKT‐Thr450 and AMPK‐Thr172 (A) Schematic showing R→K mutations in the FLAG‐PPP1CA protein. (B) In vitro methylation assays of PPP1CA mutants relative to the wild‐type PPP1CA. (C) The results of the liquid scintillation counting experiment of PPP1CA mutants relative to the wild‐type PPP1CA. (D) PFK activity assay of Carm1 OE and Ppp1ca‐KO MC3T3‐E1 and RAW264.7 cells transfected with wild‐type and mutant Ppp1ca plasmids. (E) Western blot analysis of the expression of Akt/phosphorylated Akt and osteogenesis‐related genes in Carm1‐OE and Ppp1ca‐KO MC3T3‐E1 cells transfected with wild‐type and mutant Ppp1ca plasmids. (F) Western blot analysis of the expression of AMPK/phosphorylated AMPK and osteoclast‐related genes in Carm1‐OE and Ppp1ca‐KO RAW264.7 cells transfected with wild‐type and mutant Ppp1ca plasmids. (G) The ECAR curves in Carm1‐OE and Ppp1ca‐KO MC3T3‐E1 and RAW264.7 cells transfected with wild‐type and mutant Ppp1ca plasmids treated with rotenone/antimycin A and 2‐DG (*n* = 4 independent experiments). (H) Representative images of ARS and ALP staining in Carm1‐OE and Ppp1ca‐KO MC3T3‐E1 cells transfected with wild‐type and mutant Ppp1ca plasmids after osteogenic differentiation. (I) Representative images of TRAP staining in Carm1‐OE and Ppp1ca‐KO RAW264.7 cells transfected with wild‐type and mutant Ppp1ca plasmids after osteoclast differentiation. * represents *p* < .05 vs. other groups, ** represents *p* < .01 vs. other groups.

To identify the major methylation sites of PPP1CA, we replaced three methylated arginine residues with lysine residues (Figure 6a). Although mutating all three sites suppressed methylation, mutating the R23 site dramatically decreased methylation (Figure 6b). Liquid scintillation counting results showed that mutating all three sites or mutating the R23 site caused a significant decrease in 3‐H count rates (Figure 6c).

To explore the function of PPP1CA methylation in MC3T3‐E1 and RAW264.7 cells, we employed CRISPR/Cas9 technology to KO endogenous *Ppp1ca*. To induce the OE of *Carm1*, wild‐type and mutant plasmids were transfected into cells. The PFK activity assay results demonstrated that mutating all three sites or mutating the R23 site significantly reduced enzyme activity (Figure 6d). Meanwhile, the three‐site mutations and the R23 mutation, which down‐regulated the phosphorylation levels of AKT and AMPK (Figures 6e and S9c), resulted in the down‐regulation of osteogenic‐related gene expression in MC3T3‐E1 cells and the up‐regulation of osteoclast‐related genes in RAW264.7 cells (Figures 6e, f and S9a, b). We next investigated whether PPP1CA methylation regulates metabolic fluxes, and we found that in the presence of CARM1 OE, three‐site mutations or R23 mutation decreased the rate of glycolysis in MC3T3‐E1 and RAW264.7 cells (Figure 6g). Osteogenic and osteoclastic differentiation were affected by altered cellular metabolism, and ALP activity and extracellular matrix mineralisation were significantly reduced in MC3T3‐E1 cells with three‐site mutations or R23 mutation (Figure 6h). Conversely, three‐site mutations or R23 mutation of *Ppp1ca* plasmids promoted osteoclastic differentiation of RAW264.7 cells (Figure 6i).

### 3.7 CARM1 up‐regulates PDK3 expression and reduces mitochondrial OXPHOS levels

**FIGURE 7:**
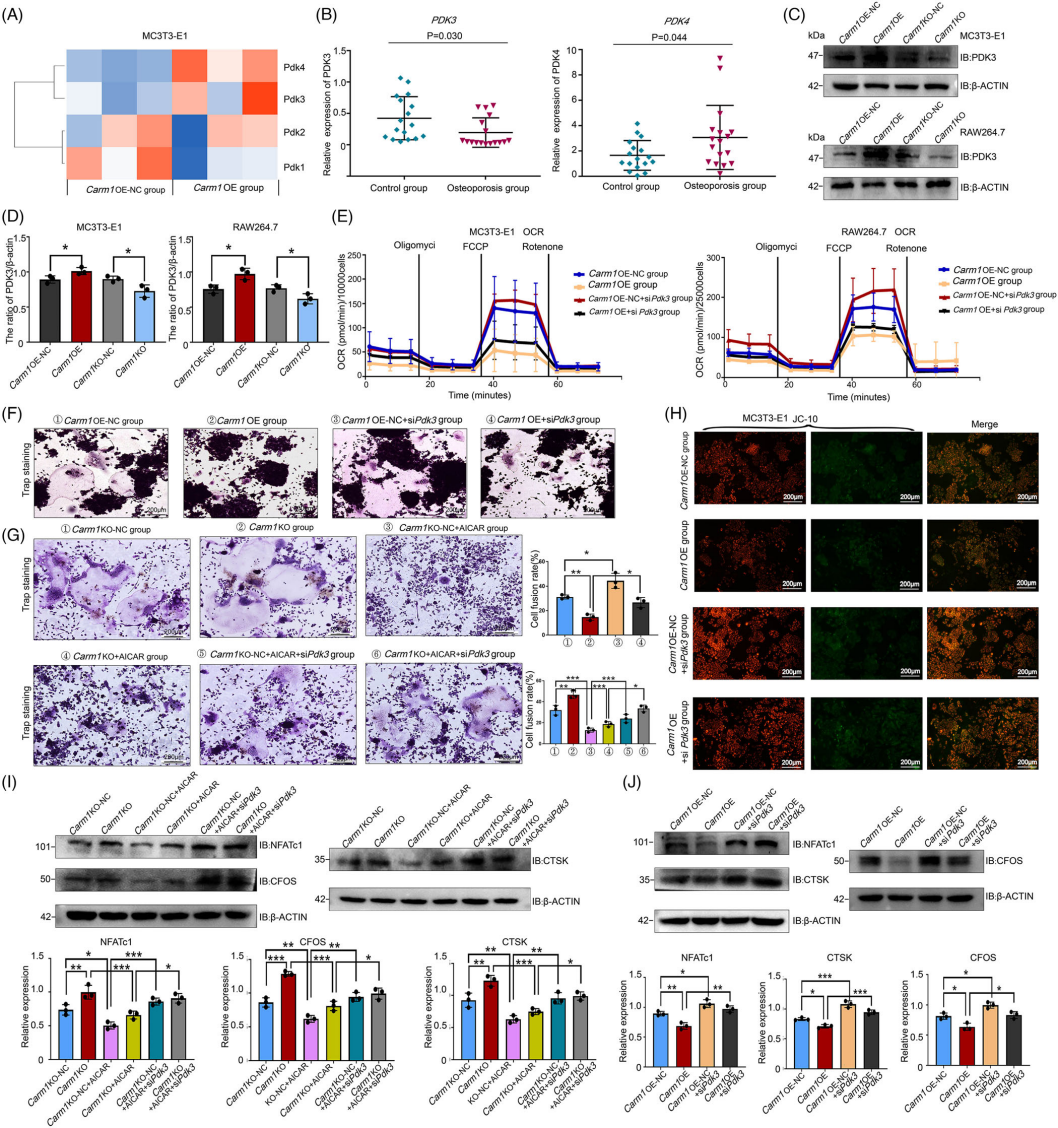
CARM1 up‐regulates PDK3 expression and reduces mitochondrial oxidative phosphorylation levels (A) heatmap of transcriptomic detection of PDK family members in Carm1‐OE and NC MC3T3‐E1 cells. (B) Quantification of PDK3 and PDK4 expression in bone samples from patients with osteoporosis. (C) Western blot analysis of PDK3 expression in Carm1‐OE and NC MC3T3‐E1 and RAW264.7 cells. (D) Quantitative data analysis of Western blot results. (E) The OCR curves in Carm1‐OE and NC cells transfected with siPdk3 with oligomycin, FCCP, and rotenone/antimycin A (*n* = 4 independent experiments). (F) Representative images of TRAP staining in Carm1‐OE and NC RAW264.7 cells transfected with siPdk3. (G) Representative images of TRAP staining in Carm1‐OE and NC RAW264.7 cells after treatment with AICAR and transfected with siPdk3. (H) Representative images of JC‐10 staining of Carm1‐OE and NC MC3T3‐E1 cells transfected with siPdk3. (I) Western blot analysis of the expression of osteoclast‐related genes in RAW264.7 cells after treatment with AICAR and transfected with siPdk3. (J) Western blot analysis of the expression of osteoclast‐related genes in Carm1‐OE and NC RAW264.7 cells after transfected with siPdk3. * represents *p* < .05 vs. other groups, ** represents *p* < .01 vs. other groups.

By analysing the transcriptomic data of MC3T3‐E1 cells, we found that the expression levels of some PDK family members were regulated by CARM1, among which the expression levels of *Pdk3* and *Pdk4* were up‐regulated, which is one of the inferred mechanisms of metabolic reprogramming (Figure 7a). RT‐PCR was used to test the expression of *PDK3* and *PDK4* in osteoporotic and control patients. It was found that *PDK3* expression was down‐regulated and *PDK4* expression was up‐regulated in osteoporotic patients (Figure 7b), suggesting that PDK3 is involved in CARM1‐mediated metabolic reprogramming. Next, we used WB to detect the expression of *Pdk3* in MC3T3‐E1, RAW264.7, mouse osteoblast and BMDM cells, and the results indicated that *Pdk3* expression was increased in the *Carm1*‐OE group (Figures 7c, d and S11a). To determine the specific mechanism by which CARM1 regulates PDK3, we first tested whether CARM1 is a transcription factor for PDK3; the results of chromatin immunoprecipitation experiments disproved this idea (Figure S10c). By searching the Cistrome Data Browser, we obtained the transcription factor of PDK3, and compared with CARM1 binding protein profile data. We found that EP300, a transcription factor of PDK3, was able to bind to CARM1, which was confirmed by Co‐IP experiments (Figure S10a) and cellular immunofluorescence staining (Figure S10b).

To investigate whether PDK3 is involved in the regulation of metabolism by CARM1, we knocked down *Pdk3* in MC3T3‐E1 and RAW264.7 cells, and the results of mitochondrial stress experiments showed that si*Pdk3* induced an increase in cell OCR (Figures 7e and S11b). Reactive oxygen species (ROS), byproducts of mitochondrial OXPHOS, are normally scavenged by nicotinamide adenine dinucleotide phosphate (NADPH) and glutathione (GSH).^45^ By knocking down *Pdk3*, cellular ROS levels were elevated, and the increase in ROS, NADP+/NADPH ratio was accompanied by a decrease in the GSH concentration (Figure S10d and e). Pyruvate dehydrogenase (PDH) catalyses the generation of pyruvate to acetyl‐CoA, and PDH is negatively regulated by PDK isoforms 1‐4.^46^, ^47^ Knockdown of *Pdk3* caused reduced PDH phosphorylation (Figures S10g and S11c), implying increased PDH activity and partially reversing the metabolic changes induced by CARM1, with increased OXPHOS fluxes and higher △*Ψ*_m_ (Figures 7h and S10f), leading to a partial reversal of the regulation of osteoclast differentiation by CARM1 (Figures 7f and S11d). Knockdown of *Pdk3* reversed the inhibition of osteoclast differentiation induced by AMPK agonist AICAR and up‐regulated the expression of osteoclast‐related genes (Figure S7g, i, and j)

## 4 DISCUSSION

**FIGURE 8:**
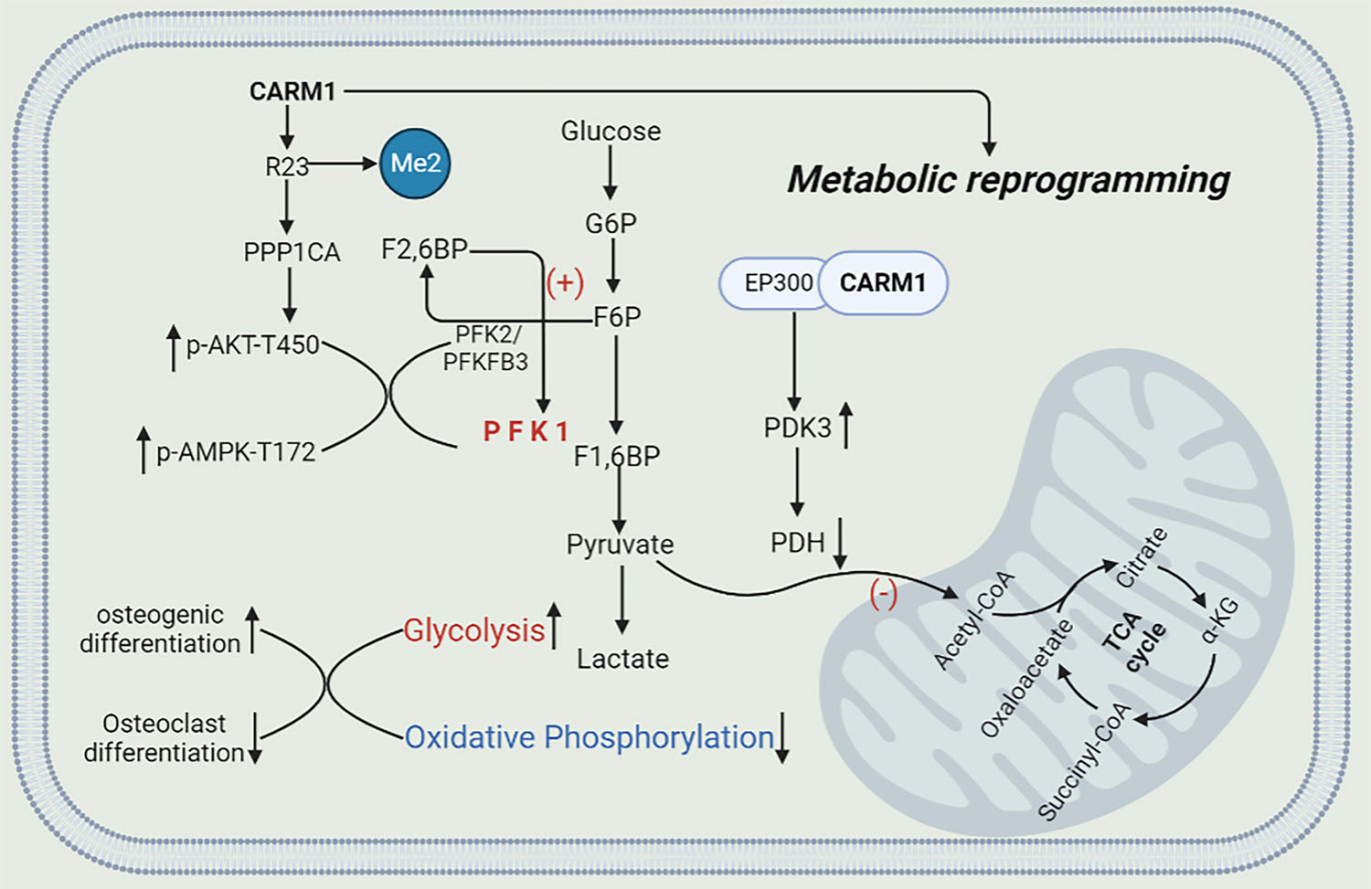
Schematic illustration describing the molecular mechanism by which CARM1 regulates osteogenic and osteoclast differentiation through metabolic reprogramming.

Bone strength depends on a delicate balance between bone formation by osteoblasts and bone resorption by osteoclasts.^48^, ^49^ The imbalance between them is an important factor leading to osteoporosis.^50^, ^51^ The simultaneous coordination of osteogenic and osteoclastic differentiation is important for the design of new treatments for osteoporosis. In this study, we found that CARM1 mediates PPP1CA R26 methylation and acts as an auxiliary coactivator to up‐regulate PDK3 expression, thereby reprogramming OXPHOS into aerobic glycolysis, a metabolic microenvironment that is more conducive to osteogenic differentiation and impaired osteoclast differentiation. Although other members of the PRMTS family also have functions that regulate metabolism, they have not been found to be dysregulated in patients with osteoporosis. Our findings reveal a novel mechanism for the simultaneous intervention of osteogenesis and osteoclast differentiation by means of metabolic regulation using the different energy requirements of osteoblasts and osteoclasts (Figure 8).

The supply, regulation and reprogramming of energy during osteogenesis and osteoblastic differentiation are complex. In addition to glycolysis and OXPHOS mentioned in this study, there are other energy supply pathways, such as glutamine catabolism, fatty acid synthesis and fatty acid oxidation.^4^, ^8^, ^52^ In our study, we focused on glucose metabolism, as it is the main form of energy production utilised by osteoblasts and osteoclasts.^1^, ^53^ Osteoclasts need a large amount of energy provided by OXPHOS in the process of differentiation and fusion.^54^ Therefore, CARM1 has a significant effect on preventing osteoclast differentiation by regulating the energy metabolism of osteoclasts. Although osteoclasts are not unique in their use of energy and mature osteoclasts have been shown to have a higher rate of glycolysis when they perform bone resorption,^54^ the application of metabolic regulation to prevent their differentiation can achieve the goal of controlling osteoporosis. The use of aerobic glycolysis by osteoblasts to generate energy differs from that of cancer cells in that they do not need to use large amounts of energy to support proliferation but rather apply metabolic intermediates to synthesise extracellular matrix proteins (e.g., collagen) required for differentiation.^55^

There have been several reports on the regulation of CARM1 metabolism in tumour cells.^21^, ^36^, ^56^ Liu et al. found that pyruvate kinase isoform M2 (PKM2) methylation by CARM1 activates aerobic glycolysis to promote the growth of breast cancer. This study suggests that PKM2 methylation has little effect on pyruvate kinase (PK) activity and that the nonglycolytic function of PKM2 regulates aerobic glycolysis rather than PK activity. This is quite different from the mechanism by which we found that PFK activity is regulated by CARM1. Wang showed that methylation of MDH1 by CARM1 inhibited glutamine metabolism in pancreatic cancer cells. This suggests that although CARM1 mediates metabolic reprogramming, the mechanisms by which CARM1 regulates metabolism may differ across diseases and cells.

Our study found that the interaction with PPP1CA is an important way for CARM1 to regulate kinase activity and thus participate in the regulation of metabolism. PPP1CA is one of the core subunits of PP1 for dephosphorylation.^57^ The specificity of PP1 for the basic motifs adjacent to the phosphorylation site is due to the inherent nature of the catalytic subunit.^58^ PPP1CA dephosphorylates AKT‐Thr450^44^ and AMPK‐Thr172.^59^ We observed that when R23 was methylated by CARM1, the dephosphorylation of PPP1CA was attenuated, and the phosphorylation levels of AKT‐Thr450 and AMPK‐Thr172 were preserved, thereby continuing activation, whereas PFK responded to kinase activation. After we knocked out PPP1CA and overexpressed CARM1, the R23 mutant PPP1CA was not efficiently methylated by CARM1, and the phosphatase activity of PP1 was retained, thus partially reversing the metabolic regulatory effect of CARM1 and attenuating osteogenic differentiation. CARM1 is mainly expressed in the nucleus, and it is also expressed in the cytoplasm. We believe that the methylation of PPP1CA by CARM1 and its biological effects mainly occur in the cytoplasm, while as a transcriptional cofactor, the interaction between CARM1 and the transcription factor EP300 mainly occurs in the nucleus, thereby regulating the transcription of PDK3.

Although AMPK has been shown to negatively regulate the aerobic glycolysis of cancer cells, AMPK has a negative regulatory role in osteoclast differentiation and its activation of PFK1 and PFK2 has also been demonstrated.^22^ Our study suggests that the up‐regulation of PDK3 by CARM1 to some extent limits the effects of AMPK on glucose metabolism and osteoclast differentiation.

In animal experiments, considering the correlation between CARM1 and cancer, we did not choose to use adenovirus or adeno‐associated virus but instead used lentivirus injection in the femoral medullary cavity. OE of CARM1 in the femur of ovariectomised mice is sufficient to meet our goals for in vivo experiments. Due to the close relationship between CARM1 and cancer, future clinical applications of our research may require targeting peptides for bone formation.^60^ Our study is flawed in that it does not provide insight into the effects of PPP1CA methylation by CARM1 on the recognition and binding of phosphorylated substrate structures, and the regulation of PDK3 by CARM1 has not been studied in sufficient detail and depth.

In summary, these findings provide insights into the function of CARM1 in regulating glucose metabolism during osteogenesis and osteoclast differentiation. CARM1 up‐regulates the activity of PFK1, the key enzyme of glycolysis, by methylation of PPP1CA R23 and regulates the flux of mitochondrial OXPHOS by regulating the expression of PDK3, which is the basis of CARM1‐mediated metabolic reprogramming (Figure 8). In addition, we also provided conceptual proof that influencing the differentiation of osteoblasts and osteoclasts through metabolic regulation may represent a new feasible treatment strategy for osteoporosis.

## CONFLICT OF INTEREST STATEMENT

The authors declare that they have no conflict of interest.

## Supporting information

Supporting Information

## Data Availability

Expression data in primary osteoblasts (OBs) obtained from women with osteoporotic fractures or severe osteoarthritis were obtained from the GEO database (https://ncbi.nlm.nih.gov/geo/query/acc.cgi?acc=GSE156508). Gene expression profiling during osteoclast differentiation mediated by RANKL was obtained from the GEO database (https://ncbi.nlm.nih.gov/geo/query/acc.cgi?acc=GSE176265). The datasets generated during the current study are available in the Figureshare repository. https://doi.org/10.6084/m9.Figureshare.21518598. All data supporting the findings of this study are available from the corresponding author upon reasonable request.
