# Supplementary material for: Arginine methylation of PPP1CA by CARM1 regulates glucose metabolism and affects osteogenic differentiation and osteoclastic differentiation
Source: Clin Transl Med. 2023 Aug 30;13(9):e1369. doi: 10.1002/ctm2.1369 (PMC10468565; doi:10.1002/ctm2.1369)
Supplement: Supplementary file 1 — Supporting Information [file CTM2-13-e1369-s001.docx]

Supplementary Materials for

Arginine Methylation of PPP1CA by CARM1 Regulates Glucose Metabolism and affects osteogenic differentiation and osteoclastic differentiation

Lu Zhang, Guangjun Jiao, Yunhao You, Xiang Li, Jincheng Liu, Zhenqian Sun, Qinghui Li, Zihan Dai, Jinlong Ma, Hongming Zhou, Gang Li, Chunyang Meng Yunzhen Chen*

Correspondence to: qilucyz@yeah.net

**This file includes:**

Supplementary Methods

Supplementary Figure and Figure Legends

Supplementary Tables 1 to 5

**Supplementary Methods**

**Preparation of osteogenic induction medium**

First, complete medium was prepared, that is, α-MEM complete medium containing 1% penicillin and streptomycin mixture and 10% fetal bovine serum. Next, 50μM ascorbic acid,10mM beta-glycerol phosphate and 100nM dexamethasone were added into the complete medium.

**Preparation of osteoclast induction medium**

First, complete medium was prepared, that is, DMEM complete medium containing 1% penicillin and streptomycin mixture and 10% fetal bovine serum. Next, 100ug/mL RANKL were added into the complete medium. If it is primary mouse bone marrow mononuclear cells that need to be induced, an additional 50ug/mL macrophage colony-stimulating factor is need to be added.

**Extraction of mouse primary osteoblasts**

Cranial cap bones from C57BL/6 mice aged less than 1 week were extracted aseptically and placed in cold PBS. The attached connective tissue was removed. The bone tissue was cleaned, cut into 0.5 mm × 0.5 mm pieces and placed in a Petri dish containing H-DMEM medium. Afterwards, 0.25% trypsin 5 mL was added and incubated at 37℃ for 20 minutes. The reaction was terminated and the supernatant was discarded.10 mL of 1.0 g/L collagenase type I was added and incubated at 37℃ for 60 minutes. The supernatant was centrifuged at 1,000 r/min for 10 minutes to precipitate cells. the cells were washed with PBS for twice and resuspended in DMEM medium supplemented with 10% (v/v) fetal bovine serum, 100 U/ml penicillin, and 100 μg/mL streptomycin. The cells were inoculated into culture flasks. Culture medium was changes after 48 hours. The cells were cultured until fusion reached approximately 90%.

**Extraction of mouse bone marrow derived macrophage**

After euthanasia, the mice were sterilized in 75% ethanol for 5 minutes. The femur and tibia were extracted and placed in 75% alcohol for 5 minutes. The ethanol was washed off the surface of the bone tissue with PBS. The ends of the bone tissue were cut and the bone marrow was well flushed out with culture medium. The liquid was filtered into sterile tubes using a 70 μm cell filter and centrifuged at 1500 rpm/min for 5 min to precipitate the cells. Discarded supernatant and added erythrocyte lysate. Mixed thoroughly and left for 5 minutes. Centrifuged again and discarded the supernatant. Then cell suspensions were prepared with culture medium. Inoculated cells into culture plates. Half of the medium was replaced after 72 hours of incubation. All medium was replaced after 120 hours. After 168 hours, the cells were ready for experiments.

**Cell culture and induction of osteogenic and osteoclastic differentiation**

MC3T3-E1 and RAW264.7 cells were purchased from Zhong Qiao Xin Zhou Biotechnology Co. Ltd. (Shanghai, China). Cells were cultured and expanded with α-MEM (Gibco, USA) containing 10% FBS, 100 U/mL penicillin and 100 μg/mL streptomycin. Osteogenesis was induced by 10 mM β-glycerophosphate, 10 nmol/L dexamethasone and 50 μg/mL ascorbic acid. For Alizarin red S staining, cells were fixed in 4% paraformaldehyde for 10 minutes at room temperature, rinsed with water and stained with an Alizarin red S staining kit (Servicebio, China). A BCIP/NBT Alkaline Phosphatase Colour Development Kit (Beyotime, Shanghai, China) was used for alkaline phosphatase staining. RAW 264.7 cells were cultured under the same conditions, and 100 ng/mL RANKL was sufficient to induce osteoclast differentiation. For TRAP staining, cells were fixed with TRAP fixing solution (Solarbio, Beijing, China) for 1 min at room temperature, rinsed with water and stained with a TRAP staining kit (Solarbio, Beijing, China).

**

**

**Supplementary Fig. 1. CARM1 promotes osteoblast differentiation of MC3T3-E1cells and impairs osteoclast differentiation of RAW264.7 cells**

a. Heatmap analysis of gene expression in primary osteoblasts obtained from women undergoing hip arthroplasty for osteoporotic fracture or severe osteoarthritis (GSE156508). b. Heatmap of gene expression profiles during RANKL-mediated osteoblast differentiation (0 h vs. 48 h) (GSE176265). c. Time curve of *Ocn*, *Col1a1*, and *Spp1* expression in MC3T3-E1 *Carm1*-OE/KO and NC cells after osteogenic differentiation. d. Time curve of *Ctsk*, *Rankl*, and *Nfatc1* expression in RAW264.7 *Carm1*-OE/KO and NC cells after osteoclastic differentiation. e. Representative images of ARS and ALP staining in MC3T3-E1 *Carm1*-OE/KO and NC cells, f. Western blot analysis of osteogenesis-related gene expression. g. Western blot analysis of the expression of osteoclast-related genes. h. Quantitative data analysis of Western blot results. i. Representative images of TRAP staining in RAW264.7 *Carm1*-OE/KO and NC cells after osteoclast differentiation. j. Representative image of RAW264.7 cells after osteoclast differentiation by immunohistochemistry. Abbreviations: ** represents P < 0.01 vs. other groups, * represents P < 0.05 vs. other groups.

**

**

**Supplementary Fig. 2. Supplementary experiments on osteogenic and osteoclast differentiation**

a. Western blot analysis of CARM1 expression in MC3T3-E1 and RAW264.7 *Carm1*-OE/KO and NC cells. b. Quantitative data analysis of western blot. c. Representative image of MC3T3-E1 cells after osteoclast differentiation by immunohistochemistry. d. Representative image of RAW264.7 cells after osteoclast differentiation by immunohistochemistry. e. Quantitative data analysis of immunohistochemistry. Abbreviations: * represents P < 0.05 vs. other groups, ** represents P < 0.01 vs. other groups.





**Supplementary Fig. 3. Supplements to the in vivo experiments.**

a. Bioluminescence images of mice after the intramedullary injection of osteoblast specific promoter lentivirus. b. Representative micro-CT images of trabecular bone from the femoral metaphysis of mice from the sham-operated group, NC group and *Carm1*-OE group. c. Trabecular bone mineral density (BMD, g/cm3), cancellous bone volume (BV/TV, %), trabecular thickness (Tb.Th), total cross-sectional cortical bone area (B.Ar), trabecular number (Tb.N), trabecular separation (Tb.Sp) and d cortical porosity (Ct. Po) were determined by micro-CT analysisb. d. Quantification analysis of CARM1 expression in bone samples from mice. e. Haematoxylin-eosin (H&E) staining of femurs from the metaphysis of mice from the sham-operated group, NC group and *Carm1*-OE group. f. Immunohistochemical staining of osteogenesis-related genes in mouse femur sections. g. Immunohistochemical staining of osteoclast -related genes in mouse femur sections. Abbreviations: * represents P < 0.05 vs. other groups, ** represents P < 0.01 vs. other groups.

**
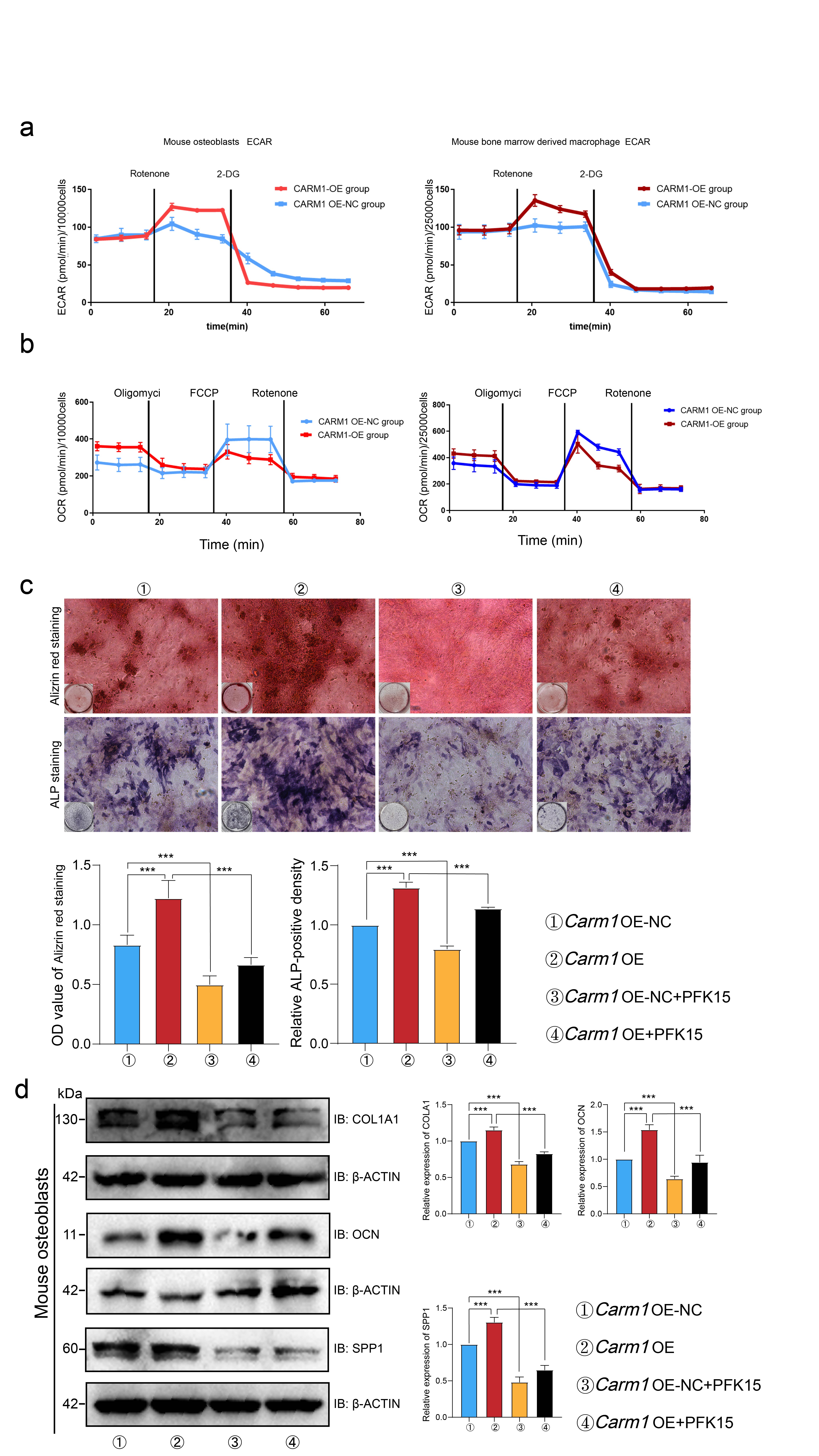
**

**Supplementary Fig. 4. *Carm1* mediates metabolic reprogramming in** **mouse osteoblasts and BMDM**

a. The ECAR curves in *Carm1*-OE and NC cells treated with rotenone/antimycin A and 2-DG. b. The OCR curves in *Carm1*-OE and NC cells treated with oligomycin, FCCP, and rotenone/antimycin A. c. Representative images of ARS and ALP staining in mouse osteoblasts after treatment with PFK15. f. Western blot analysis of the expression of osteogenesis related genes in mouse osteoblasts after treatment with PFK15. Abbreviations: * represents P < 0.05 vs. other groups, ** represents P < 0.01 vs. other groups. *** represents P < 0.001 vs. other groups.

**
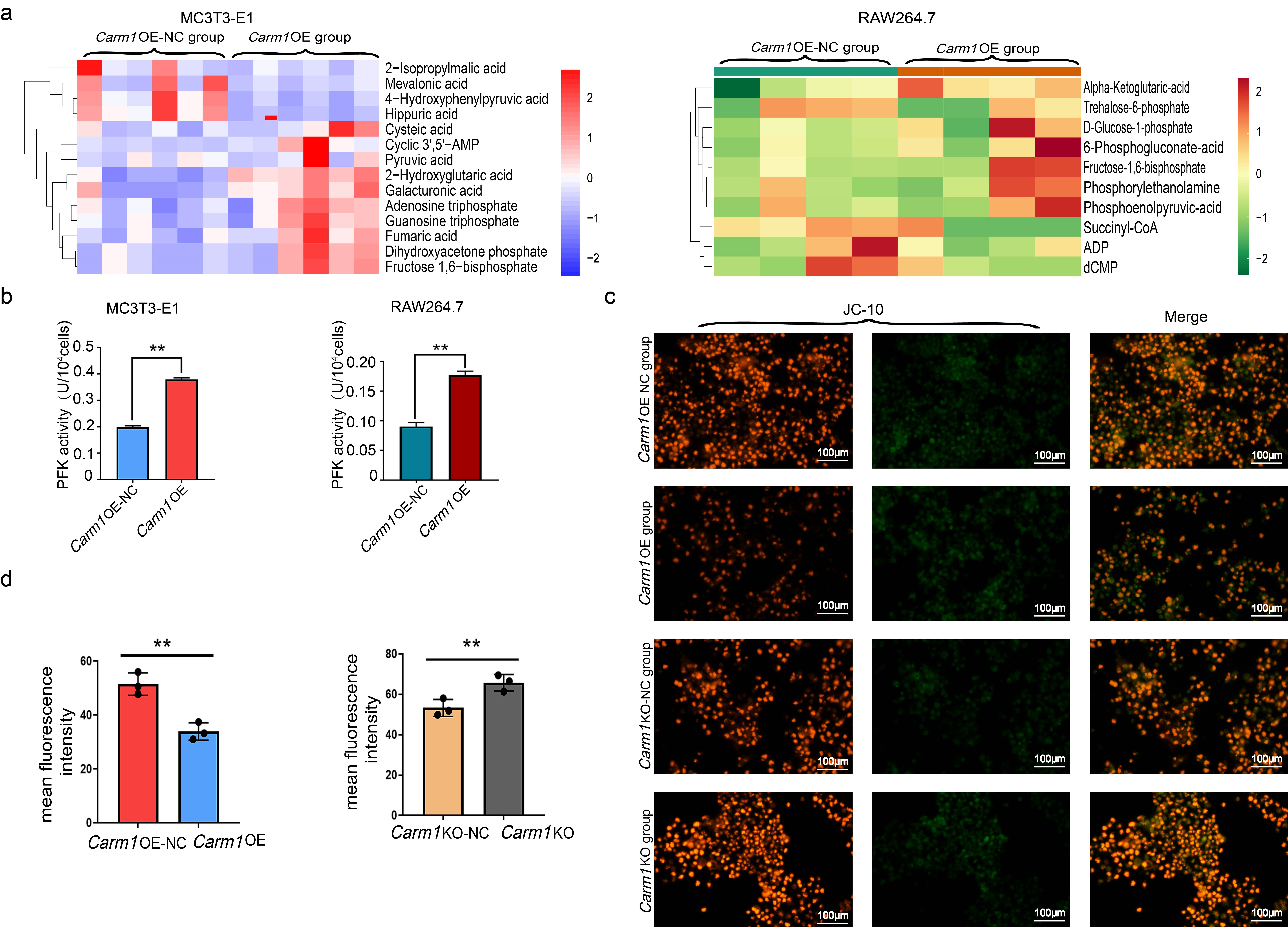
**

**Supplementary Fig. 5 Supplementary experiments on the regulation of PFK1 and PFKFB3 activities by CARM1**

a. Heatmap of metabolites with differences in metabolomic detection of MC3T3-E1 cells, and RAW264.7 cells. b. PFK activity assay of *Carm1*-OE and NC MC3T3-E1 and RAW264.7 cells. c. Representative images of JC-10 staining of *Carm1*-OE/KO and NC cells. d. Quantitative data analysis of JC-10results. Abbreviations: * represents P < 0.05 vs. other groups, ** represents P < 0.01 vs. other groups.

**

**

**Supplementary Fig. 6. Supplementary experiments on the regulation of PFK activity by AKT and AMPK**

a. Heatmap of transcriptomic analysis in *Carm1*-OE and NC MC3T3-E1 cells. b. Western blot analysis of phospho-PFK1/PKFKB3 expression in MC3T3-E1 and RAW264.7 cells. *Carm1*-OE/KO and NC cells c. Quantitative data analysis of Western blot results. d. Representative images of ARS and ALP staining in MC3T3-E1 cells after treatment with MK2206. e. Western blot analysis of the expression of osteogenesis-related genes in MC3T3-E1 cells after treatment with MK2206. f. Quantitative data analysis of Western blot results. Abbreviations: * represents P < 0.05 vs. other groups, ** represents P < 0.01 vs. other groups, *** represents P < 0.001 vs. other groups.


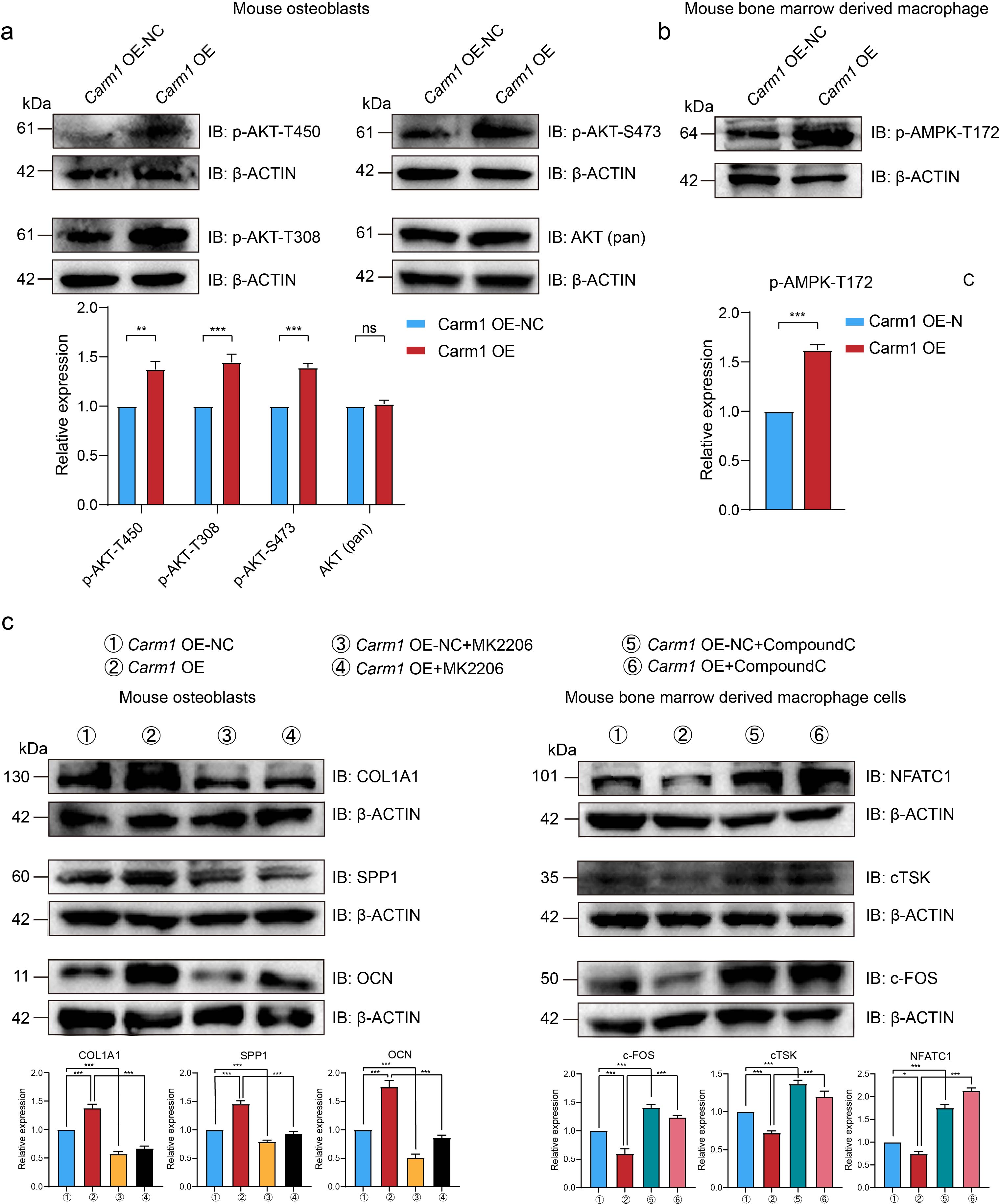


**Supplementary Fig. 6. Supplementary experiments on the regulation of PFK activity by AKT and AMPK in primary cells**

a. Western blot analysis of AKT and phosphorylated AKT expression in mouse osteoblasts *Carm1*-OE and NC cells. b. western blot analysis of expression of AKT/AMPK and phosphorylated AKT/AMPK in BMDM *Carm1*-OE /KO and NC cells. c. Western blot analysis of the expression of osteogenesis-related genes in mouse osteoblasts and BMDM after treatment with MK2206 or Compound C. Abbreviations: * represents P < 0.05 vs. other groups, ** represents P < 0.01 vs. other groups, *** represents P < 0.001 vs. other groups.

**
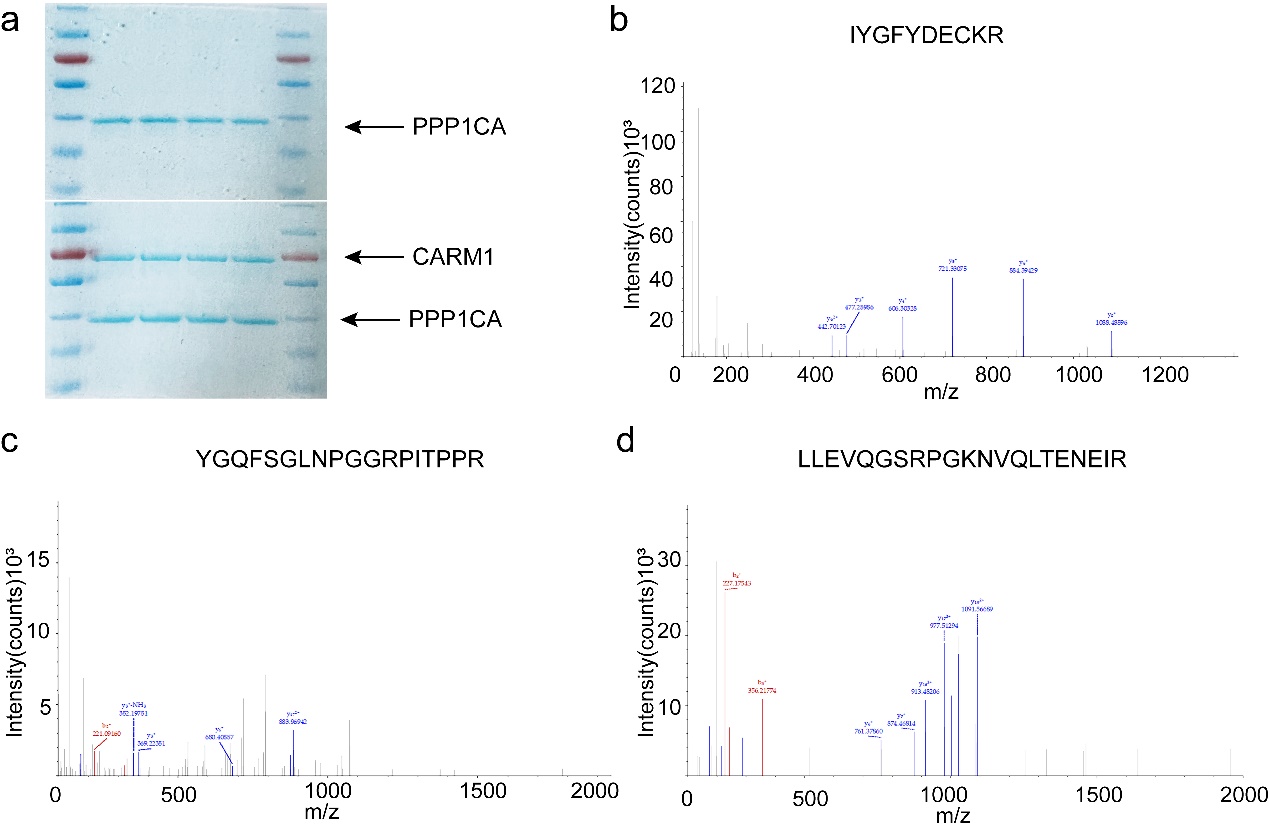
**

**Supplementary Fig. 8. Supplementary experiments on the in vitro methylation detection of PPP1CA**

a. Image of sample protein after in vitro methylation reaction, electrophoresis, Coomassie staining, and decolorization. b. Image of PPP1CA R23 dimethylation mass spectrometry. c. Image of PPP1CA R142 methylation mass spectrometry. d. Image of PPP1CA R317 methylation mass spectrometry.

**

**

**Supplementary Fig. 9. Quantitative data from the PPP1CA R23 mutation assay**

a. Quantitative data analysis of Western blot results in *Carm1*-OE and *Ppp1ca*-KO MC3T3-E1 cells transfected with wild-type and mutant *Ppp1ca* plasmids. b. Western blot analysis of AMPK expression in RAW264.7 cells, and quantitative data analysis of Western blot results in *Carm1*-OE and *Ppp1ca*-KO RAW264.7 cells transfected with wild-type and mutant *Ppp1ca* plasmids. c. Western blot analysis of the expression of Akt/phosphorylated Akt and osteogenesis-related genes in *Carm1*-OE and *Ppp1ca*-KO HEK 293-T cells transfected with wild-type and mutant Ppp1ca plasmids. Abbreviations: * represents P < 0.05 vs. other groups, ** represents P < 0.01 vs. other groups.

**

**

**Supplementary Fig. 10. Supplementary experiments on the involvement of PDK3 in CARM1-regulated metabolism**

a. Coimmunoprecipitation of CARM1 with endogenous EP300 in MC3T3-E1 and RAW264.7 cells. b. Representative images of immunofluorescence staining of EP300 and CARM1 in MC3T3-E1 and RAW264.7 cells. c. Chromatin immunoprecipitation assay results. d. Representative image of reactive oxygen species detection in *Carm1*-OE and NC cells transfected with si*Pdk3.* e. Quantitative data analysis of NADP+/NADPH ratio and GSH concentration. f. Representative images of JC-10 staining of *Carm1*-OE and NC RAW264.7 cells transfected with si*Pdk3*. g. Western blot analysis of the expression of PDH and phospho-PDH in *Carm1*-OE and NC cells transfected with si*Pdk3*, and quantitative data analysis of western blot results. Abbreviations: * represents P < 0.05 vs. other groups, ** represents P < 0.01 vs. other groups.

**
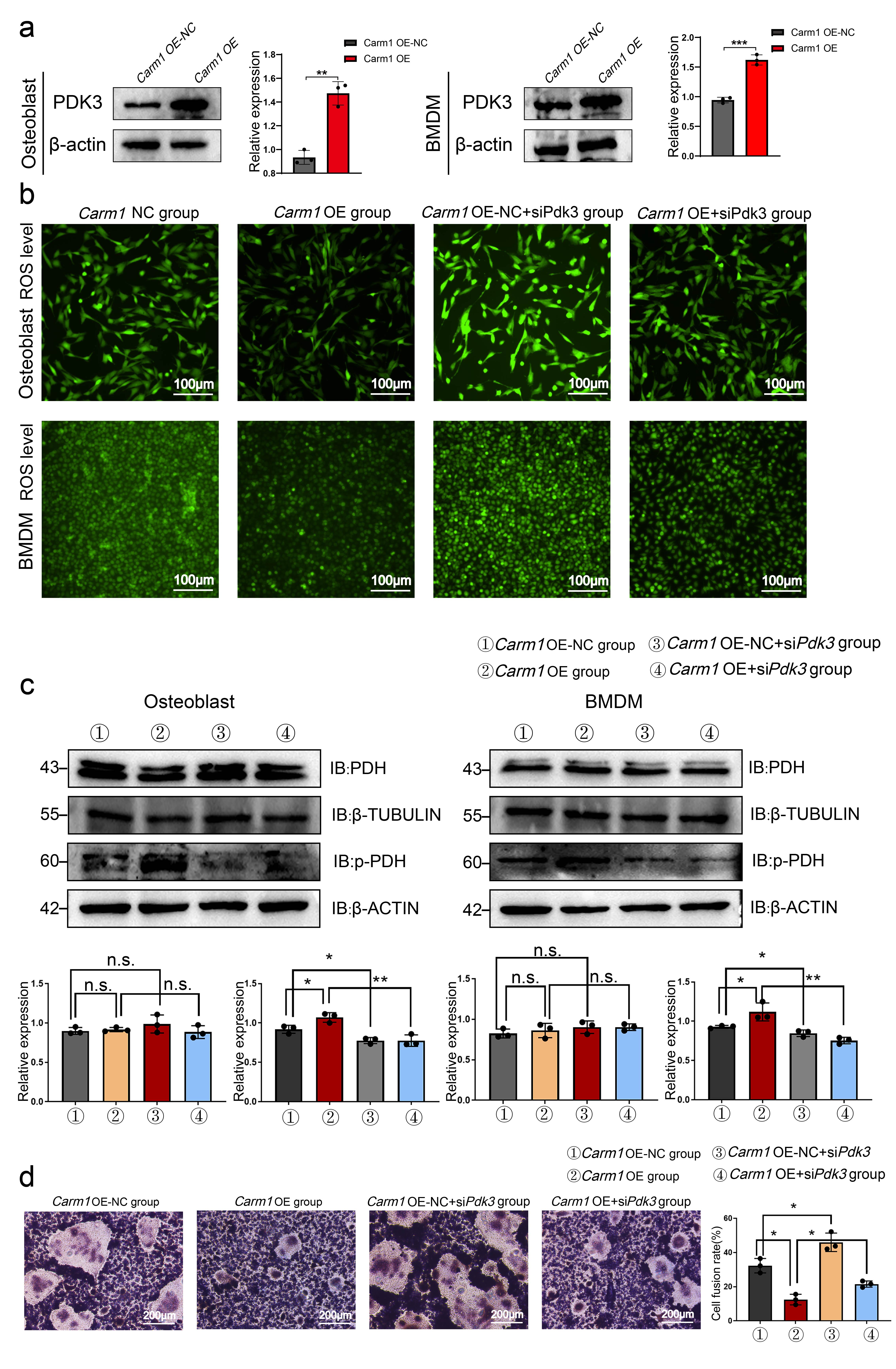
**

**Supplementary Fig. 11. Supplementary experiments on the involvement of PDK3 in CARM1-regulated metabolism in primary cells**

a. Western blot analysis of PDK3 expression in *Carm1*-OE and NC mouse osteoblasts and BMDM cells. b. Representative image of reactive oxygen species detection in Carm1-OE and NC cells transfected with siPdk3. c. Western blot analysis of the expression of PDH and phospho-PDH in *Carm1*-OE and NC cells transfected with si*Pdk3*, and quantitative data analysis of western blot results. d. Representative images of TRAP staining in *Carm1*-OE and NC RAW264.7 cells transfected with si*Pdk3*.

**Supplementary Tables 1 Proteins interacting with CARM1 in 293T cells**

This table comes from Dr. Liu's research. (Liu F, Ma F, Wang Y, et al. PKM2 methylation by CARM1 activates aerobic glycolysis to promote tumorigenesis. Nat Cell Biol. 2017. 19(11): 1358-1370).

| ***Antibody*** | ***Catalog Number*** | | ***Manufacturer*** |
| --- | --- | --- | --- |
| A6NFM2 | PYCR1 | HUMAN Pyrroline-5-carboxylate reductase | 1.11E-16 |
| Q86X55 | CARM1 | HUMAN Histone-arginine methyltransferase CARM1 | 6.66E-15 |
| B7ZAT2 | CCT2 | HUMAN Chaperonin containing TCP1, subunit 2 (Beta), isoform CRA_c | 2.44E-14 |
| B1ANR0 | PABPC4 | HUMAN Poly(A) binding protein, cytoplasmic 4 (Inducible form) | 3.11E-14 |
| P54886 | ALDH18A1 | HUMAN Delta-1-pyrroline-5-carboxylate synthase | 6.88E-14 |
| B4DUU6 | PKM2 | HUMAN Pyruvate kinase | 3.89E-12 |
| P26599 | PTBP1 | HUMAN Polypyrimidine tract-binding protein 1 | 1.25E-11 |
| B7Z2F4 | CCT4 | HUMAN T-complex protein 1 subunit delta | 1.76E-11 |
| E9PFU1 | E9PFU1 | HUMAN Uncharacterized protein | 3.04E-11 |
| P08238 | HSP90AB1 | HUMAN Heat shock protein HSP 90-beta | 5.20E-11 |
| Q9UBS4 | DNAJB11 | HUMAN DnaJ homolog subfamily B member 11 | 6.22E-11 |
| Q07021 | C1QBP | HUMAN Complement component 1 Q subcomponent-binding protein, mitochondrial | 6.28E-11 |
| B3KXI2 | SEC23A | HUMAN Uncharacterized protein | 7.20E-11 |
| O14929 | HAT1 | HUMAN Histone acetyltransferase type B catalytic subunit | 8.49E-11 |
| C9JGC2 | CPSF6 | HUMAN Uncharacterized protein | 8.97E-11 |
| Q12905 | ILF2 | HUMAN Interleukin enhancer-binding factor 2 | 1.01E-10 |
| P22695 | UQCRC2 | HUMAN Cytochrome b-c1 complex subunit 2, mitochondrial | 1.07E-10 |
| E9PC52 | RBBP7 | HUMAN Uncharacterized protein | 1.55E-10 |
| B4DFF1 | HNRNPK | HUMAN Uncharacterized protein | 1.77E-10 |
| B1ALA9 | PRPS1 | HUMAN Phosphoribosyl pyrophosphate synthetase 1 | 1.78E-10 |
| P09874 | PARP1 | HUMAN Poly [ADP-ribose] polymerase 1 | 3.00E-10 |
| O43809 | NUDT21 | HUMAN Cleavage and polyadenylation specificity factor subunit | 3.65E-10 |
| A5A3E0 | POTEF | HUMAN POTE ankyrin domain family member F | 4.18E-10 |
| D6R938 | CAMK2D | HUMAN Calcium/calmodulin-dependent protein kinase (CaM kinase) II delta, isoform CRA_e | 4.45E-10 |
| A8K854 | hCG_1983504 | HUMAN HCG1983504, isoform CRA_f | 4.48E-10 |
| E7EM48 | MCM4 | HUMAN Uncharacterized protein | 4.59E-10 |
| P22626 | HNRNPA2B1 | HUMAN Heterogeneous nuclear ribonucleoproteins A2/B1 | 4.68E-10 |
| P49411 | TUFM | HUMAN Elongation factor Tu, mitochondrial | 7.52E-10 |
| E9PJ04 | SF3B2 | HUMAN Uncharacterized protein | 8.99E-10 |
| B4DUT7 | GMPS | HUMAN Uncharacterized protein | 9.73E-10 |
| Q9UMS4 | PRPF19 | HUMAN Pre-mRNA-processing factor 19 | 9.74E-10 |
| A6NG51 | SPTAN1 | HUMAN Uncharacterized protein | 1.01E-09 |
| C9J2Z7 | EIF4G1 | HUMAN Uncharacterized protein | 1.09E-09 |
| P30050 | RPL12 | HUMAN 60S ribosomal protein L12 | 1.13E-09 |
| E9PCY4 | DCAF7 | HUMAN Uncharacterized protein | 1.16E-09 |
| B4E2T8 | CANX | HUMAN Uncharacterized protein | 1.34E-09 |
| HIST2H2BF | HIST2H2BF | HUMAN Histone H2B | 1.40E-09 |
| B4DYD8 | CCT5 | HUMAN Uncharacterized protein | 1.41E-09 |
| GNB2L1 | GNB2L1 | HUMAN Uncharacterized protein | 1.74E-09 |
| B7Z4T9 | CCT7 | HUMAN Chaperonin containing TCP1, subunit 7 (Eta), isoform CRA_c | 1.85E-09 |
| F8W6I0 | EEF1A1 | HUMAN Elongation factor 1-alpha | 1.97E-09 |
| P40222 | TXLNA | HUMAN Alpha-taxilin | 2.41E-09 |
| P11021 | HSPA5 | HUMAN 78 kDa glucose-regulated protein | 2.62E-09 |
| Q01082 | SPTBN1 | HUMAN Spectrin beta chain, brain 1 | 3.28E-09 |
| A8MZ91 | PRMT5 | HUMAN Protein arginine methyltransferase 5, isoform CRA_d | 3.71E-09 |
| Q9BTD8 | RBM42 | HUMAN RNA-binding protein 42 | 3.84E-09 |
| A8K7X6 | PCBP2 | HUMAN Poly(RC) binding protein 2, isoform CRA_b | 3.84E-09 |
| C9J5V9 | YBX1 | HUMAN Uncharacterized protein | 3.92E-09 |
| P04181 | OAT | HUMAN Ornithine aminotransferase, mitochondrial | 4.80E-09 |
| P46782 | RPS5 | HUMAN 40S ribosomal protein S5 | 5.26E-09 |
| C9JLI6 | RPS27L | HUMAN Ribosomal protein S27 | 5.83E-09 |
| O15067 | PFAS | HUMAN Phosphoribosylformylglycinamidine synthase | 5.83E-09 |
| P08670 | VIM | HUMAN Vimentin | 6.02E-09 |
| E7EU96 | CSNK2A1 | HUMAN Uncharacterized protein | 6.32E-09 |
| B4E102 | EIF4A1 | HUMAN Uncharacterized protein | 6.74E-09 |
| A8K1R6 | ATXN2L | HUMAN Uncharacterized protein | 7.20E-09 |
| B7ZAR1 | CCT5 | HUMAN Uncharacterized protein | 7.55E-09 |
| E7EP96 | KHSRP | HUMAN Uncharacterized protein | 7.55E-09 |
| A8MXQ4 | LDHA | HUMAN L-lactate dehydrogenase | 8.38E-09 |
| B4DFK9 | HNRNPH2 | HUMAN Uncharacterized protein | 8.78E-09 |
| F8VRV5 | DYNLL1 | HUMAN Uncharacterized protein | 1.27E-08 |
| P0CG12 | CHTF8 | HUMAN Chromosome transmission fidelity protein 8 homolog isoform 2 | 1.41E-08 |
| B1N949 | PPFIA4 | HUMAN Liprin alpha4 | 1.44E-08 |
| B2ZZ83 | FLNB | HUMAN Filamin B | 1.46E-08 |
| E9PKD5 | PSMC3 | HUMAN Uncharacterized protein | 1.69E-08 |
| O94776 | MTA2 | HUMAN Metastasis-associated protein MTA2 | 1.99E-08 |
| F5GZG3 | EEF2 | HUMAN Uncharacterized protein | 2.04E-08 |
| P13489 | RNH1 | HUMAN Ribonuclease inhibitor | 2.06E-08 |
| Q8NC51 | SERBP1 | HUMAN Plasminogen activator inhibitor 1 RNA-binding protein | 2.40E-08 |
| E7EUD8 | PYCR2 | HUMAN Uncharacterized protein | 2.56E-08 |
| P07900 | HSP90AA1 | HUMAN Heat shock protein HSP 90-alpha | 2.62E-08 |
| E7ERF2 | TCP1 | HUMAN T-complex protein 1 subunit alpha | 2.90E-08 |
| SERA | PHGDH | HUMAN D-3-phosphoglycerate dehydrogenase | 2.91E-08 |
| B4E363 | FARSA | HUMAN Uncharacterized protein | 3.21E-08 |
| B4DZT4 | SEC24C | HUMAN Uncharacterized protein | 3.45E-08 |
| P52732 | KIF11 | HUMAN Kinesin-like protein KIF11 | 3.71E-08 |
| E9PGK3 | TUBA1C | HUMAN Uncharacterized protein | 3.72E-08 |
| B4DHI8 | EPB41L2 | HUMAN Uncharacterized protein | 3.85E-08 |
| B4E3P0 | ACLY | HUMAN Uncharacterized protein | 4.01E-08 |
| B2R603 | HNRNPC | HUMAN Uncharacterized protein | 4.54E-08 |
| B4E1T7 | PPP2R2A | HUMAN Uncharacterized protein | 4.67E-08 |
| C9JMU5 | DDX17 | HUMAN Uncharacterized protein | 4.76E-08 |
| P07954 | FUMH | HUMAN Fumarate hydratase, mitochondrial | 4.79E-08 |
| F8VPE8 | RPLP0 | HUMAN Uncharacterized protein | 5.37E-08 |
| P30041 | PRDX6 | HUMAN Peroxiredoxin-6 | 5.51E-08 |
| A6NIT8 | HNRNPL | HUMAN Uncharacterized protein | 6.57E-08 |
| A8MWI8 | CCT7 | HUMAN Uncharacterized protein | 6.96E-08 |
| F5H442 | TSG101 | HUMAN Uncharacterized protein | 7.23E-08 |
| F5H370 | EEF2 | HUMAN Uncharacterized protein | 7.87E-08 |
| F5H3Y8 | SRRT | HUMAN Uncharacterized protein | 7.92E-08 |
| B4DN87 | SERPINH1 | HUMAN Uncharacterized protein | 8.90E-08 |
| E9PP73 | COPB1 | HUMAN Uncharacterized protein | 9.88E-08 |
| P31930 | UQCRC1 | HUMAN Cytochrome b-c1 complex subunit 1, mitochondrial | 1.15E-07 |
| P62244 | RPS15A | HUMAN 40S ribosomal protein S15a | 1.29E-07 |
| B4DGB4 | PLS3 | HUMAN Uncharacterized protein | 1.30E-07 |
| P31689 | DNAJA1 | HUMAN DnaJ homolog subfamily A member 1 | 1.38E-07 |
| A8MTG3 | ZNF207 | HUMAN Uncharacterized protein | 1.49E-07 |
| B4DUR9 | AHSA1 | HUMAN Uncharacterized protein | 1.53E-07 |
| RPIA | RPIA | HUMAN Ribose-5-phosphate isomerase | 1.55E-07 |
| P62258 | YWHAE | HUMAN 14-3-3 protein epsilon | 1.61E-07 |
| TBAL3 | TUBAL3 | HUMAN Tubulin alpha chain-like 3 | 1.81E-07 |
| Q99615 | DNAJC7 | HUMAN DnaJ homolog subfamily C member 7 | 1.82E-07 |
| O43684 | BUB3 | HUMAN Mitotic checkpoint protein BUB3 | 1.91E-07 |
| E9PJZ7 | PPFIA1 | HUMAN Uncharacterized protein | 2.17E-07 |
| Q9BXP5 | SRRT | HUMAN Serrate RNA effector molecule homolog | 2.40E-07 |
| P62913 | RPL11 | HUMAN 60S ribosomal protein L11 | 2.53E-07 |
| A6NE14 | CCT3 | HUMAN Uncharacterized protein | 2.65E-07 |
| C9IZD2 | POLD2 | HUMAN Uncharacterized protein | 2.80E-07 |
| DDX17 | DDX17 | HUMAN Isoform 4 of Probable ATP-dependent RNA helicase DDX17 | 2.85E-07 |
| B3KSH1 | EIF3F | HUMAN HCG1784554, isoform CRA_a | 2.87E-07 |
| E9PD78 | RRM1 | HUMAN Ribonucleoside-diphosphate reductase | 3.11E-07 |
| NUDC | NUDC | HUMAN Nuclear migration protein nudC | 3.28E-07 |
| C9JZW3 | EEF1B2 | HUMAN Uncharacterized protein | 3.75E-07 |
| B4DKS8 | HNRNPF | HUMAN Uncharacterized protein | 3.90E-07 |
| E7ETA0 | YBX1 | HUMAN Uncharacterized protein | 4.19E-07 |
| A6NIZ1 | RP1BL | HUMAN Ras-related protein Rap-1b-like protein | 4.21E-07 |
| A6NKV8 | PAIP1 | HUMAN Uncharacterized protein | 4.23E-07 |
| B4DMC6 | EEF2 | HUMAN Uncharacterized protein | 4.37E-07 |
| HNRPK | HNRNPK | HUMAN Heterogeneous nuclear ribonucleoprotein K | 4.41E-07 |
| B0AZV0 | STRAP | HUMAN Uncharacterized protein | 4.71E-07 |
| B4DR91 | PSMC6 | HUMAN Uncharacterized protein | 5.17E-07 |
| B4DUA5 | P4HB | HUMAN Uncharacterized protein | 5.53E-07 |
| D6R904 | TPM3 | HUMAN Uncharacterized protein | 5.61E-07 |
| H4 | HIST1H4A | HUMAN Histone H4 | 5.68E-07 |
| E7EQG2 | EIF4A2 | HUMAN Uncharacterized protein | 5.81E-07 |
| F8W0P7 | ATP5B | HUMAN Uncharacterized protein | 6.30E-07 |
| Q15427 | SF3B4 | HUMAN Splicing factor 3B subunit 4 | 7.31E-07 |
| A6NNR3 | PPP1CA | HUMAN Serine/threonine-protein phosphatase | 7.35E-07 |
| E7EQR6 | TCP1 | HUMAN T-complex protein 1 subunit alpha | 7.75E-07 |
| MCM7 | MCM7 | HUMAN DNA replication licensing factor MCM7 | 8.22E-07 |
| E7EWU2 | FLNA | HUMAN Uncharacterized protein | 8.57E-07 |
| C9J6D7 | KIAA0664 | HUMAN Uncharacterized protein | 8.95E-07 |
| E7EX81 | NCL | HUMAN Uncharacterized protein | 9.75E-07 |
| B5MEG9 | MAP4 | HUMAN Uncharacterized protein | 9.81E-07 |
| Q92945 | KHSRP | HUMAN Far upstream element-binding protein 2 | 1.06E-06 |
| Q13263 | TRIM28 | HUMAN Transcription intermediary factor 1-beta | 1.11E-06 |
| A6NDY9 | FLNA | HUMAN Filamin A | 1.14E-06 |
| B7Z6M1 | PLS3 | HUMAN Uncharacterized protein | 1.16E-06 |
| Q5TH29 | NDRG3 | HUMAN NDRG family member 3 (Fragment) | 1.35E-06 |
| P10809 | HSPD1 | HUMAN 60 kDa heat shock protein, mitochondrial | 1.36E-06 |
| B4DP38 | WDR77 | HUMAN Uncharacterized protein | 1.59E-06 |
| F5H897 | TRAP1 | HUMAN Uncharacterized protein | 1.66E-06 |
| P26196 | DDX6 | HUMAN Probable ATP-dependent RNA helicase DDX6 | 1.68E-06 |
| A8MXT5 | SAFB2 | HUMAN Uncharacterized protein | 1.72E-06 |
| C9J8K3 | PRRC2A | HUMAN Uncharacterized protein | 1.80E-06 |
| E7EPW1 | GAPDH | HUMAN Uncharacterized protein | 1.88E-06 |
| P12236 | SLC25A6 | HUMAN ADP/ATP translocase 3 | 1.97E-06 |
| P05141 | SLC25A5 | HUMAN ADP/ATP translocase 2 | 1.97E-06 |
| E9PMH2 | AIP | HUMAN Uncharacterized protein | 1.99E-06 |
| Q9NPH2 | ISYNA1 | HUMAN Inositol-3-phosphate synthase 1 | 2.03E-06 |
| B3KQ25 | PSME3 | HUMAN Uncharacterized protein | 2.11E-06 |
| B4DV12 | UBB | HUMAN Uncharacterized protein | 2.13E-06 |
| Q5H928 | HSD17B10 | HUMAN Hydroxysteroid (17-beta) dehydrogenase 10 | 2.21E-06 |
| F8VQY6 | RPLP0 | HUMAN Uncharacterized protein | 2.23E-06 |
| Q06830 | PRDX1 | HUMAN Peroxiredoxin-1 | 2.39E-06 |
| A6NEM2 | HCFC1 | HUMAN Uncharacterized protein | 2.82E-06 |
| E7ES38 | SEC23A | HUMAN Uncharacterized protein | 2.97E-06 |
| Q00610 | CLTC | HUMAN Clathrin heavy chain 1 | 2.99E-06 |
| E9PLW6 | AASDHPPT | HUMAN Uncharacterized protein | 3.09E-06 |
| C9J1F6 | PRRC2A | HUMAN Uncharacterized protein | 3.17E-06 |
| F5H634 | PHGDH | HUMAN Uncharacterized protein | 3.18E-06 |
| P0C7P4 | UQCRFS1P1 | HUMAN Putative cytochrome b-c1 complex subunit Rieske-like protein 1 | 3.46E-06 |
| A8K092 | ATP5A1 | HUMAN ATP synthase subunit alpha | 4.19E-06 |
| F5H039 | GPHN | HUMAN Uncharacterized protein | 4.26E-06 |
| B4DPJ8 | CCT6A | HUMAN Uncharacterized protein | 4.34E-06 |
| B7Z5C0 | DNAJA1 | HUMAN Uncharacterized protein | 4.82E-06 |
| A6NG23 | PRRC2A | HUMAN Uncharacterized protein | 4.84E-06 |
| F5GWT9 | PFAS | HUMAN Uncharacterized protein | 4.85E-06 |
| B4DDR3 | API5 | HUMAN Uncharacterized protein | 5.16E-06 |
| C9JRV9 | UBA5 | HUMAN Uncharacterized protein | 5.32E-06 |
| B4DDF7 | PPP2R1A | HUMAN Uncharacterized protein | 5.46E-06 |
| Q7Z784 | DNAJC7 | HUMAN DNAJC7 protein | 6.01E-06 |
| P04908 | HIST1H2AB | HUMAN Histone H2A type 1-B/E | 6.06E-06 |
| B4DKP9 | EIF4A1 | HUMAN Uncharacterized protein | 6.46E-06 |
| B3KSF1 | CACYBP | HUMAN Uncharacterized protein | 6.60E-06 |
| Q8TDX7 | NEK7 | HUMAN Serine/threonine-protein kinase Nek7 | 6.69E-06 |
| F5H721 | WBP11 | HUMAN Uncharacterized protein | 6.91E-06 |
| B7Z8M7 | RAB1A | HUMAN Uncharacterized protein | 6.93E-06 |
| E9PGZ0 | CSDE1 | HUMAN Uncharacterized protein | 7.48E-06 |
| P14866 | HNRNPL | HUMAN Heterogeneous nuclear ribonucleoprotein L | 7.71E-06 |
| P41250 | GARS | HUMAN Glycyl-tRNA synthetase | 7.99E-06 |
| P62304 | SNRPE | HUMAN Small nuclear ribonucleoprotein E | 8.21E-06 |
| B4DDD7 | PDHB | HUMAN Uncharacterized protein | 8.98E-06 |
| B4DLJ0 | ALDH1B1 | HUMAN Uncharacterized protein | 9.28E-06 |
| E9PBS1 | PAICS | HUMAN Uncharacterized protein | 9.37E-06 |
| A6NCD2 | CCT6A | HUMAN Uncharacterized protein | 9.48E-06 |
| Q13347 | EIF3I | HUMAN Eukaryotic translation initiation factor 3 subunit I | 9.81E-06 |
| Q15365 | PCBP1 | HUMAN Poly(rC)-binding protein 1 | 1.02E-05 |
| A2A274 | ACO2 | HUMAN Aconitase 2, mitochondrial | 1.23E-05 |
| E7ENH9 | ACLY | HUMAN Uncharacterized protein | 1.29E-05 |
| E9PF63 | ROCK2 | HUMAN Uncharacterized protein | 1.37E-05 |
| C9JHC7 | POLD2 | HUMAN Uncharacterized protein | 1.52E-05 |
| B3KRS5 | HDAC2 | HUMAN Histone deacetylase | 1.56E-05 |
| E5RG31 | MCM4 | HUMAN Uncharacterized protein | 1.62E-05 |
| D6RD67 | MCCC2 | HUMAN Uncharacterized protein | 1.78E-05 |
| Q5TCU6 | TLN1 | HUMAN Talin 1 | 2.08E-05 |
| Q13185 | CBX3 | HUMAN Chromobox protein homolog 3 | 2.27E-05 |
| P25705 | ATP5A1 | HUMAN ATP synthase subunit alpha, mitochondrial | 2.54E-05 |
| P04075 | ALDOA | HUMAN Fructose-bisphosphate aldolase A | 2.54E-05 |
| Q8IWX8 | CHERP | HUMAN Calcium homeostasis endoplasmic reticulum protein | 2.64E-05 |
| B3KX72 | HNRNPU | HUMAN Uncharacterized protein | 2.67E-05 |
| C9JTC6 | LRRFIP1 | HUMAN Uncharacterized protein | 2.68E-05 |
| B8ZZB3 | CCDC141 | HUMAN Putative uncharacterized protein FLJ39502 | 2.74E-05 |
| B7Z5T2 | CRB1 | HUMAN Uncharacterized protein | 2.99E-05 |
| P63173 | RPL38 | HUMAN 60S ribosomal protein L38 | 3.22E-05 |
| P14625 | HSP90B1 | HUMAN Endoplasmin | 3.41E-05 |
| F8VPV9 | ATP5B | HUMAN ATP synthase subunit beta | 3.49E-05 |
| B7Z1N6 | ALDOC | HUMAN Fructose-bisphosphate aldolase | 3.76E-05 |
| E7EPT2 | SRRT | HUMAN Uncharacterized protein | 3.98E-05 |
| B9ZVR1 | MAP4 | HUMAN Microtubule-associated protein | 4.00E-05 |
| Q9NRJ5 | PAPOLB | HUMAN Poly(A) polymerase beta | 4.86E-05 |
| E5RGW4 | NPM1 | HUMAN Uncharacterized protein | 4.89E-05 |
| Q5JYA8 | MTHFD1L | HUMAN Methylenetetrahydrofolate dehydrogenase (NADP+ dependent) 1-like (Fragment) | 5.05E-05 |
| E9PCN9 | KPNA3 | HUMAN Uncharacterized protein | 5.06E-05 |
| P12004 | PCNA | HUMAN Proliferating cell nuclear antigen | 5.18E-05 |
| P60842 | EIF4A1 | HUMAN Eukaryotic initiation factor 4A-I | 5.75E-05 |
| E9PNG0 | AMPD2 | HUMAN Uncharacterized protein | 6.41E-05 |
| P62249 | RPS16 | HUMAN 40S ribosomal protein S16 | 6.54E-05 |
| P06753 | TPM3 | HUMAN Tropomyosin alpha-3 chain | 6.66E-05 |
| B4DDN4 | EIF3C | HUMAN Uncharacterized protein | 6.99E-05 |
| D6RBJ6 | CENPH | HUMAN Uncharacterized protein | 7.32E-05 |
| Q96LL3 | C16orf92 | HUMAN Uncharacterized protein C16orf92 | 7.38E-05 |
| P35555 | FBN1 | HUMAN Fibrillin-1 | 7.77E-05 |
| B3KNT8 | NAP1L1 | HUMAN Uncharacterized protein | 7.78E-05 |
| P04183 | TK1 | HUMAN Thymidine kinase, cytosolic | 8.17E-05 |
| Q96F45 | ZNF503 | HUMAN Zinc finger protein 503 | 8.21E-05 |
| E7ET15 | U2SURP | HUMAN Uncharacterized protein | 8.46E-05 |
| Q14683 | SMC1A | HUMAN Structural maintenance of chromosomes protein 1A | 8.94E-05 |
| B4DYZ8 | CCDC108 | HUMAN Uncharacterized protein | 9.24E-05 |
| P08912 | CHRM5 | HUMAN Muscarinic acetylcholine receptor M5 | 9.33E-05 |
| Q9P217 | ZSWIM5 | HUMAN Zinc finger SWIM domain-containing protein 5 | 9.63E-05 |
| P49327 | FASN | HUMAN Fatty acid synthase | 1.04E-04 |
| E7ESU4 | NAT10 | HUMAN Uncharacterized protein | 1.05E-04 |
| P50990 | CCT8 | HUMAN T-complex protein 1 subunit theta | 1.08E-04 |
| B3KUF4 | GATA4 | HUMAN Uncharacterized protein | 1.15E-04 |
| C9J5E5 | HDLBP | HUMAN Uncharacterized protein | 1.18E-04 |
| P28069 | POU1F1 | HUMAN Pituitary-specific positive transcription factor 1 | 1.18E-04 |
| Q92793 | CREBBP | HUMAN CREB-binding protein | 1.22E-04 |
| P49792 | RANBP2 | HUMAN E3 SUMO-protein ligase RanBP2 | 1.28E-04 |
| E7ES42 | QSER1 | HUMAN Uncharacterized protein | 1.31E-04 |
| P22102 | GART | HUMAN Trifunctional purine biosynthetic protein adenosine-3 | 1.36E-04 |
| P36551 | CPOX | HUMAN Coproporphyrinogen-III oxidase, mitochondrial | 1.36E-04 |
| P38919 | EIF4A3 | HUMAN Eukaryotic initiation factor 4A-III | 1.37E-04 |
| B7Z7T5 | USP7 | HUMAN Uncharacterized protein | 1.41E-04 |
| P12270 | TPR | HUMAN Nucleoprotein TPR | 1.41E-04 |
| Q15437 | SEC23B | HUMAN Protein transport protein Sec23B | 1.45E-04 |
| Q14444 | CAPRIN1 | HUMAN Caprin-1 | 1.49E-04 |
| P06753-2 | TPM3 | HUMAN Isoform 2 of Tropomyosin alpha-3 chain | 1.57E-04 |
| Q00839 | HNRNPU | HUMAN Heterogeneous nuclear ribonucleoprotein U | 1.68E-04 |
| E7EP00 | SEC24C | HUMAN Uncharacterized protein | 1.74E-04 |
| Q07954 | LRP1 | HUMAN Prolow-density lipoprotein receptor-related protein 1 | 1.79E-04 |
| P00492 | HPRT1 | HUMAN Hypoxanthine-guanine phosphoribosyltransferase | 1.81E-04 |
| Q14204 | DYNC1H1 | HUMAN Cytoplasmic dynein 1 heavy chain 1 | 1.81E-04 |
| B7ZAV6 | KPNB1 | HUMAN Uncharacterized protein | 1.89E-04 |
| D6RGZ2 | THOC3 | HUMAN Uncharacterized protein | 1.89E-04 |
| Q02750 | MAP2K1 | HUMAN Dual specificity mitogen-activated protein kinase kinase 1 | 1.94E-04 |
| F5GWQ6 | CAPN6 | HUMAN Uncharacterized protein | 1.99E-04 |
| E7EV07 | ARHGEF4 | HUMAN Uncharacterized protein | 2.09E-04 |
| Q8WXK1 | ASB15 | HUMAN Ankyrin repeat and SOCS box protein 15 | 2.09E-04 |
| Q6NSI8 | KIAA1841 | HUMAN Uncharacterized protein KIAA1841 | 2.15E-04 |
| E7ER27 | HSD17B4 | HUMAN Uncharacterized protein | 2.16E-04 |
| Q99961 | SH3GL1 | HUMAN Endophilin-A2 | 2.28E-04 |
| Q7Z6J4-2 | FGD2 | HUMAN Isoform 2 of FYVE, RhoGEF and PH domain-containing protein 2 | 2.35E-04 |
| Q92734 | TFG | HUMAN Protein TFG | 2.41E-04 |
| O95486 | SEC24A | HUMAN Protein transport protein Sec24A | 2.55E-04 |
| B7ZKM8 | SEC24B | HUMAN SEC24B protein | 2.55E-04 |
| B1AKL3 | EIF4ENIF1 | HUMAN Eukaryotic translation initiation factor 4E nuclear import factor 1 | 2.57E-04 |
| Q1KMD3 | HNRNPUL2 | HUMAN Heterogeneous nuclear ribonucleoprotein U-like protein 2 | 2.75E-04 |
| D6RIT2 | HNRNPH1 | HUMAN Uncharacterized protein | 2.93E-04 |
| Q14315 | FLNC | HUMAN Filamin-C | 2.96E-04 |
| A5YVE9 | PDHA1 | HUMAN Mitochondrial PDHA1 | 2.98E-04 |
| F8W6U1 | MYO18A | HUMAN Uncharacterized protein | 2.99E-04 |
| Q09472 | EP300 | HUMAN Histone acetyltransferase p300 | 3.01E-04 |
| C9JYS8 | NONO | HUMAN Uncharacterized protein | 3.11E-04 |
| E7EQS9 | C22orf28 | HUMAN Uncharacterized protein | 3.11E-04 |
| C9JLF8 | CHAF1A | HUMAN Uncharacterized protein | 3.11E-04 |
| B7Z3Q6 | GARNL3 | HUMAN Uncharacterized protein | 3.11E-04 |
| B4E0K9 | MAN2B1 | HUMAN Uncharacterized protein | 3.25E-04 |
| O95347 | SMC2 | HUMAN Structural maintenance of chromosomes protein 2 | 3.33E-04 |
| Q09028 | RBBP4 | HUMAN Histone-binding protein RBBP4 | 3.35E-04 |
| E7EP25 | IFT172 | HUMAN Uncharacterized protein | 3.49E-04 |
| Q86U70 | LDB1 | HUMAN LIM domain-binding protein 1 | 3.50E-04 |
| Q06124 | PTPN11 | HUMAN Tyrosine-protein phosphatase non-receptor type 11 | 3.60E-04 |
| O60673 | REV3L | HUMAN DNA polymerase zeta catalytic subunit | 3.71E-04 |
| B4DUR8 | CCT3 | HUMAN Uncharacterized protein | 3.75E-04 |
| B4DDB4 | CDC45 | HUMAN Uncharacterized protein | 3.86E-04 |
| F5GWE6 | CPSF7 | HUMAN Uncharacterized protein | 4.08E-04 |
| Q58FF6 | HSP90AB4P | HUMAN Putative heat shock protein HSP 90-beta 4 | 4.09E-04 |
| C9JFC3 | MAP4 | HUMAN Uncharacterized protein | 4.21E-04 |
| Q9NUU7 | DDX19A | HUMAN ATP-dependent RNA helicase DDX19A | 4.24E-04 |
| Q5JYJ7 | MCF2 | HUMAN MCF.2 cell line derived transforming sequence | 4.24E-04 |
| E7ESI9 | ABCA12 | HUMAN Uncharacterized protein | 4.39E-04 |
| Q5JTM7 | OLFML2A | HUMAN Olfactomedin-like 2A (Fragment) | 4.42E-04 |
| E9PC44 | SEC24D | HUMAN Uncharacterized protein | 4.52E-04 |
| A8MW50 | LDHB | HUMAN L-lactate dehydrogenase | 4.63E-04 |
| Q9Y6A5 | TACC3 | HUMAN Transforming acidic coiled-coil-containing protein 3 | 4.67E-04 |
| B4DZI8 | COPB2 | HUMAN Coatomer protein complex, subunit beta 2 (Beta prime), isoform CRA_b | 4.83E-04 |
| A8MTU8 | SREBF1 | HUMAN Uncharacterized protein | 4.91E-04 |
| F8VP90 | MMP14 | HUMAN Uncharacterized protein | 5.14E-04 |
| B4DST5 | PTPN23 | HUMAN Uncharacterized protein | 5.17E-04 |
| F5H1G8 | GNA13 | HUMAN Uncharacterized protein | 5.24E-04 |
| C9JJP5 | TFG | HUMAN Uncharacterized protein | 5.31E-04 |
| O00231 | PSMD11 | HUMAN 26S proteasome non-ATPase regulatory subunit 11 | 5.40E-04 |
| Q15717 | ELAVL1 | HUMAN ELAV-like protein 1 | 5.43E-04 |
| B7Z406 | PPARGC1A | HUMAN Uncharacterized protein | 5.45E-04 |
| Q9C0D5 | TANC1 | HUMAN Protein TANC1 | 5.59E-04 |
| B3KXF2 | KIAA0368 | HUMAN Uncharacterized protein | 5.60E-04 |
| A6NN01 | H2AFV | HUMAN Histone H2A | 5.66E-04 |
| F5H047 | CPSF7 | HUMAN Uncharacterized protein | 5.83E-04 |
| C9J634 | PDHB | HUMAN Uncharacterized protein | 5.84E-04 |
| Q9NZP6 | C15orf2 | HUMAN Protein C15orf2 | 5.87E-04 |
| F5H315 | XAB2 | HUMAN Uncharacterized protein | 5.91E-04 |
| B4DRZ3 | PTPRE | HUMAN Uncharacterized protein | 6.11E-04 |
| Q9HCN6-3 | GP6 | HUMAN Isoform 3 of Platelet glycoprotein VI | 6.22E-04 |
| A8K6S2 | KIFC3 | HUMAN Kinesin family member C3, isoform CRA_c | 6.23E-04 |
| F5H2F4 | MTHFD1 | HUMAN Uncharacterized protein | 6.40E-04 |
| Q5T0K0 | C1orf49 | HUMAN Chromosome 1 open reading frame 49 | 6.45E-04 |
| Q13464 | ROCK1 | HUMAN Rho-associated protein kinase 1 | 6.50E-04 |
| C9JRR9 | CASP2 | HUMAN Uncharacterized protein | 6.59E-04 |
| Q9HCJ3 | RAVER2 | HUMAN Ribonucleoprotein PTB-binding 2 | 6.60E-04 |
| O60312 | ATP10A | HUMAN Probable phospholipid-transporting ATPase VA | 6.75E-04 |
| Q8NBF2 | NHLRC2 | HUMAN NHL repeat-containing protein 2 | 6.77E-04 |
| Q9NXG2 | THUMPD1 | HUMAN THUMP domain-containing protein 1 | 6.78E-04 |
| Q9UBV8 | PEF1 | HUMAN Peflin | 6.80E-04 |
| A6NF00 | LAMA2 | HUMAN Uncharacterized protein | 6.85E-04 |
| E9PHY5 | EPB41L2 | HUMAN Uncharacterized protein | 7.01E-04 |
| B4DR47 | ZC3H12C | HUMAN Uncharacterized protein | 7.25E-04 |
| A6NKC6 | RP1L1 | HUMAN Uncharacterized protein | 7.41E-04 |
| Q9UPU7 | TBC1D2B | HUMAN TBC1 domain family member 2B | 7.59E-04 |
| Q6NX45 | ZNF774 | HUMAN Zinc finger protein 774 | 7.74E-04 |
| B7Z6B6 | ZNF346 | HUMAN Uncharacterized protein | 7.82E-04 |
| P08621 | SNRNP70 | HUMAN U1 small nuclear ribonucleoprotein 70 kDa | 8.07E-04 |
| B4DFM5 | ATP6V1B2 | HUMAN Uncharacterized protein | 8.24E-04 |
| B4DME8 | DDX1 | HUMAN Uncharacterized protein | 8.46E-04 |
| B7Z8R3 | HMGCS2 | HUMAN Uncharacterized protein | 8.72E-04 |
| C9JYC9 | ZMYM2 | HUMAN Uncharacterized protein | 8.75E-04 |
| O43303 | CCP110 | HUMAN Centriolar coiled-coil protein of 110 kDa | 9.11E-04 |
| C9JJE2 | SF1 | HUMAN Uncharacterized protein | 9.18E-04 |
| Q7Z4P5 | GDF7 | HUMAN Growth/differentiation factor 7 | 9.27E-04 |
| Q9Y3V2-3 | RWDD3 | HUMAN Isoform 3 of RWD domain-containing protein 3 | 9.32E-04 |
| A8MWX9 | DCTN1 | HUMAN Uncharacterized protein | 9.53E-04 |
| Q8NGN1 | OR6T1 | HUMAN Olfactory receptor 6T1 | 9.75E-04 |
| Q9Y5L4 | TIMM13 | HUMAN Mitochondrial import inner membrane translocase subunit Tim13 | 1.54E-06 |
| Q99767 | APBA2 | HUMAN Amyloid beta A4 precursor protein-binding family A member 2 | 5.51E-06 |
| Q9BST9 | RTKN | HUMAN Rhotekin | 1.30E-04 |
| Q32MZ4 | LRRFIP1 | HUMAN Leucine-rich repeat flightless-interacting protein 1 | 1.44E-04 |
| O15245 | SLC22A1 | HUMAN Solute carrier family 22 member 1 | 2.29E-04 |
| B4DDG1 | UBE2L3 | HUMAN Ubiquitin carrier protein | 6.67E-04 |
| Q76KP1 | B4GALNT4 | HUMAN N-acetyl-beta-glucosaminyl-glycoprotein 4-beta-N-acetylgalactosaminyltransferase 1 | 8.36E-04 |
| Q04656 | ATP7A | HUMAN Copper-transporting ATPase 1 | 8.75E-04 |
| P32456 | GBP2 | HUMAN Interferon-induced guanylate-binding protein 2 | 9.21E-04 |
| A6NM15 | CBWD7 | HUMAN Putative COBW domain-containing protein 7 | 9.66E-04 |

**Supplementary Tables 2 List of primary antibodies**

| ***Antibody*** | ***Catalog Number*** | ***Manufacturer*** | ***RRID*** |
| --- | --- | --- | --- |
| *Anti-CARM1 antibody* | *Ab243638* | *Abcam* |  |
| *CARM1 mouse mAb* | *sc-393381* | *Santa Cruz* | *AB_2732840* |
| *Anti-AKT1 (phosphoT450) antibody* | *ab108266* | *Abcam* | *AB_10862734* |
| *Anti-PFKFB3 (phosphoS461) antibody* | *ab202291* | *Abcam* |  |
| *Anti-Cathepsin K antibody* | *ab187647* | *Abcam* | *AB_2891139* |
| *Phospho-Akt (Ser473) Rabbit mAb* | *4060* | *Cell Signaling Technology* | *AB_2315049* |
| *Phospho-Akt (Thr308) Rabbit mAb* | *4056* | *Cell Signaling Technology* | *AB_331163* |
| *Akt (pan) Rabbit mAb* | *4691* | *Cell Signaling Technology* | *AB_915783* |
| *NFAT2 (D15F1) Rabbit mAb* | *8032* | *Cell Signaling Technology* | *AB_10829466* |
| *Pyruvate Dehydrogenase Rabbit mAb* | *3205* | *Cell Signaling Technology* | *AB_2162926* |
| *PPP1CA Rabbit pAb* | *A12468* | *ABclonal* | *AB_2759312* |
| *Mouse anti DDDDK-Tag mAb* | *AE005* | *ABclonal* | *AB_2770401* |
| *Osteocalcin (BGLAP) Rabbit mAb* | *A20800* | *ABclonal* |  |
| *Anti-AMPK alpha2 Antibody* | *bs-2771R* | *Bioss* | *AB_10857967* |
| *Anti-phosphoAMPK(Thr172)Antibody* | *bs-4002R* | *Bioss* | *AB_10855790* |
| *Anti-phosphoPFK1(Ser775) Antibody* | *bs-16457R* | *Bioss* | *AB_2924870* |
| *Anti-phospho-PDH (Ser293) Antibody* | *bs-4036R* | *Bioss* | *AB_10856439* |
| *Rabbit Anti-Ep300 antibody* | *bs-6954R* | *Bioss* | *AB_2924871* |
| *Bone Alkaline Phosphatase Antibody* | *bs-6292R* | *Bioss* | *AB_11067005* |
| *Rabbit Anti-Collagen I antibody* | *bs-7158R* | *Bioss* |  |
| *osteopontin/OPN/SPP1 Antibody* | *sc-21742* | *Santa Cruz* | *AB_2194997* |
| *Rabbit Anti-PDK3 Antibody* | *bs-12599R* | *Bioss* |  |
| *Rabbit Anti-FLAG Tag (CT) Antibody* | *bs-0879R* | *Bioss* | *AB_10857922* |
| *Rabbit Anti-beta-Actin Antibody* | *bs-0061R* | *Bioss* | *AB_10855480* |
| *Rabbit Anti-Beta tubulin Antibody* | *bs-4511R* | *Bioss* | *AB_11114300* |
| *c-Fos Antibody* | *AF5354* | *Affinity* | *AB_2837839* |
| *RANKL Antibody* | *AF0313* | *Affinity* | *AB_2833477* |

**Supplementary Tables 3 Nucleotide sequences of q-PCR primers**

| ***Names*** | ***Species*** | ***Sequence (5′ to 3′)*** |
| --- | --- | --- |
| *CARM1* | *Homo sapiens* | Sense: CCTCATCCAGTTCGCCACA  Antisense: GCTGGGACAGGTAGCCATAAA |
| *β-ACTIN* | *Homo sapiens* | Sense: TGACGTGGACATCCGCAAAG  Antisense: CTGGAAGGTGGACAGCGAGG |
| *PDK3* | *Homo sapiens* | Sense: CGCTCTCCATCAAACAATTCCT  Antisense: CCACTGAAGGGCGGTTAAGTA |
| *PDK4* | *Homo sapiens* | Sense: GGTGGTGTTCCCCTGAGAAT  Antisense: GCAAGCCGTAACCAAAACCA |
| *Carm1* | *Mus musculus* | Sense: AACCCCTTCTTCAGGTACACAG  Antisense: AGGTCGTAGGCAGTAGGCAT |
| *Alpl* | *Mus musculus* | Sense: GCACCTGCCTTACCAACTCT  Antisense: TGGAGACGCCCATACCATC |
| *Spp1* | *Mus musculus* | Sense: CACATGAAGAGCGGTGAGTCT  Antisense: CCTTTCCGTTGTTGTCCTG |
| *Ocn* | *Mus musculus* | Sense: TCTGACCTCACAGATGCCAAG  Antisense: AGGGTTAAGCTCACACTGCT |
| *Nfatc1* | *Mus musculus* | Sense: GGAGAGTCCGAGAATCGAGAT  Antisense: TTGCAGCTAGGAAGTACGTCT |
| *Rankl* | *Mus musculus* | Sense: AGCCGAGACTACGGCAAGTA  Antisense: AAAGTACAGGAACAGAGCGATG |
| *Ctsk* | *Mus musculus* | Sense: CTCGGCGTTTAATTTGGGAGA  Antisense: TCGAGAGGGAGGTATTCTGAGT |
| β*-actin* | *Mus musculus* | Sense: GGCTGTATTCCCCTCCATCG  Antisense: CCAGTTGGTAACAATGCCATGT |

**Supplementary Tables 4 Sequences of siRNA**

| ***Names*** | ***Species*** | ***Sequence (5′ to 3′)*** |
| --- | --- | --- |
| *siPDK3* | *Mus musculus* | S: GGACUUCGGAAGGGAUAAUTT  AS: AUUAUCCCUUCCGAAGUCCTT |

**Supplementary Tables 5 Patient general data**

| **Control group** | **Osteoporosis group** | |
| --- | --- | --- |
| Number of patients:17(11 female 6mle) | | Number of patients:17(11 female 6mle) P＞0.05 |
| Age:61.35±7.85 | | Age:64.00±5.85 P＞0.05 |
